# Enhanced microfluidic multi-target separation by positive and negative magnetophoresis

**DOI:** 10.1038/s41598-024-64330-y

**Published:** 2024-06-10

**Authors:** Saud Khashan, Abdulkarem A. Odhah, Marwan Taha, Anas Alazzam, Mohamed Al-Fandi

**Affiliations:** 1 Department of Mechanical Engineering, Jordan University of Science and Technology, Irbid, 22110 Jordan; 2 System on Chip Lab, Department of Mechanical and Nuclear Engineering, Khalifa University of Science & Technology, 127788 Abu Dhabi, United Arab Emirates

**Keywords:** Magnetophoresis, Separation, Microfluidics, Continuous separation, Magnetic separation, Biomedical engineering, Medical research

## Abstract

We introduce magnetophoresis-based microfluidics for sorting biological targets using positive Magnetophoresis (pM) for magnetically labeled particles and negative Magnetophoresis (nM) for label-free particles. A single, externally magnetized ferromagnetic wire induces repulsive forces and is positioned across the focused sample flow near the main channel's closed end. We analyze magnetic attributes and separation performance under two transverse dual-mode magnetic configurations, examining magnetic fields, hydrodynamics, and forces on microparticles of varying sizes and properties. In pM, the dual-magnet arrangement (DMA) for sorting three distinct particles shows higher magnetic gradient generation and throughput than the single-magnet arrangement (SMA). In nM, the numerical results for SMA sorting of red blood cells (RBCs), white blood cells (WBCs), and prostate cancer cells (PC3-9) demonstrate superior magnetic properties and throughput compared to DMA. Magnetized wire linear movement is a key design parameter, allowing device customization. An automated device for handling more targets can be created by manipulating magnetophoretic repulsion forces. The transverse wire and magnet arrangement accommodate increased channel depth without sacrificing efficiency, yielding higher throughput than other devices. Experimental validation using soft lithography and 3D printing confirms successful sorting and separation, aligning well with numerical results. This demonstrates the successful sorting and separating of injected particles within a hydrodynamically focused sample in all systems. Both numerical and experimental findings indicate a separation accuracy of 100% across various Reynolds numbers. The primary channel dimensions measure 100 µm in height and 200 µm in width. N52 permanent magnets were employed in both numerical simulations and experiments. For numerical simulations, a remanent flux density of 1.48 T was utilized. In the experimental setup, magnets measuring 0.5 × 0.5 × 0.125 inches and 0.5 × 0.5 × 1 inch were employed. The experimental data confirm the device's capability to achieve 100% separation accuracy at a Reynolds number of 3. However, this study did not explore the potential impact of increased flow rates on separation accuracy.

## Introduction

Miniaturization, currently a prevailing trend in many technologies, has driven the production of microdevices to take advantage of microscale phenomena that cannot be achieved with macroscale systems and processes. Lab-on-a-chip (LOC), used in many biotechnological applications to perform fast and portable analysis of biological and chemical samples, is an important example of such microsystems. The advancement of LOCs relies heavily on the intelligent utilization of many favorable micro-scale phenomena of the fluids and species transported through microchannels. Research on microfluidics focused on studying transport phenomena in fluids and species within microchannels and chambers and the methods used to create these microstructures^1^. The separation of target biological entities from samples using magnetophoretic separation is increasingly becoming practical for many diagnostic and therapeutic applications. In magnetophoretic separation, the sample is exposed to an external magnetic field, which creates forces on the magnetizable components due mainly to the magnetic field gradient. This method provides a contactless and harmless application to the biological cells during separation^2^.

Several applications of magnetophoresis have been explored, particularly in cell filtration and blood cell separation^3,4^. Early efforts were made by Chalmers et al.^5^ to quantify labeled cells using magnetic susceptibility, while Han et al.^6^ advanced this work with a more sophisticated implementation. Han et al. developed a theoretical model for a microfluidic separator, leading to the creation of a diamagnetic micro-separator^7,8^. This device utilized induced incremental magnetic gradients from a ferromagnetic wire, which was invasively magnetized and positioned beneath the microchannel in alignment with the flow direction^6^. In another study, Lawson et al.^9^ examined the dynamics of particles drawn towards a magnetized wire in the presence of non-negligible gravitational forces and a broad spectrum of Stokes numbers. After these pioneering works, there have been significant advancements in the performance and efficiency of axial repulsive-based separation devices.

A comparative study was performed by Wu et al. for Halbach and alternating magnetic arrays to investigate the repulsive forces experienced by nonmagnetic beads^10^. As expected, the magnetic arrays' sorting capability decreased when the flow rate increased. The performance of the magnetic arrays was optimized by studying the distance between the microchannel and a Halbach array^11^ and by varying the locations and number of external magnets and the ferrofluid sheath streams^12^. Offset permanent magnets were used in other works to focus nonmagnetic particles in negative magnetophoretic systems as a replacement for sheath flows^13,14^. The separation efficiency could be enhanced by integrating a Nickel sputtering with the chip substrate^15^ or employing a micromixer before the separation region to shorten the sample preparation time^16^.

The performance of separation devices can also be improved by combining passive inertial focusing and active magnetophoretic separation^17^. Kim et al.^18^ presented a two-part separation device combined with a narrow microchannel that lines up particles and a permanent external magnet that deflects them laterally, depending on their size. Zhou et al.^19^ introduced a proof-of-concept sheathless microfluidic device that combines flow-induced inertial lift and ferrofluid-induced magnetic force to sort diamagnetic particles by size. Du et al.^20^ studied an inertial-based separator combined with a nonmagnetic bead sorter and analyzed the impact of magnetic pole direction, microchannel shape, and inlet flow speed on the bead's deflection. Shiriny et al.^21^ investigated a spiral microchannel sorter coupled with a separation device that utilizes a Halbach array and dual streams to enhance the focusing of particles in the microchannel. Zhou and Wang^22^ employed the interface of co-flowing ferrofluid streams and the sample. The magnetic field was generated by injecting neodymium powder into a prefabricated microstructure in the device. The same principle was applied by Chen et al.^23^, who used an inertial co-flow for ferrofluid and water to re-suspend the separated particles from the carrying ferrofluid.

Further investigations on the same area were studied by Xue et al.^24^ using a numerical study for the separation of sub-microdiamagnetic particles. Zhou et al.^25^ utilized U-shaped microchannels to enhance particle separation in ferrofluids. The same strategy was used to separate peanut-shaped and spherical particles of the same volume^26^. Hybridization between the dielectrophoretic DEP and the magnetophoretic (MAP) separation methods could also improve performance. Low and Kadri^27^ developed a computational model to study the integration of a magnetophoretic platform with a DEP-based system to separate circulating tumor cells. Shamloo et al.^28^ conducted a numerical study of a two-step micro separator device that uses MAP physics to separate red blood cells (RBCs) and platelets (PLTs) in the first step, followed by the DEP technique to separate white blood cells (WBCs) and circulating tumor cells (CTCs) in the second step. In a recent numerical study by Tran Thi et al.^29^, a coupled MAP-DEP system was combined with a hydrodynamic focus to improve performance.

Several works^30–35^ used EMG 408, a commercial water-based ferrofluid, diluted with de-ionized water as the medium due to its biocompatibility and optical properties^36^. However, other studies used modified or custom-made ferrofluids for various purposes. Shen et al.^37^ increased the magnetic susceptibility of the flowing medium by adding gadolinium-diethylenetriamine pentaacetic acid (Gd-DTPA), which improved repulsive forces in the separation process and enhanced the biocompatibility of the ferrofluid^38^. Adding Polyethylene oxide (PEO) to the ferrofluid increased viscoelasticity and improved the focusing of particles before separation^39,40^. Paramagnetic manganese (II) chloride medium utilized at different concentrations to separate nonmagnetic particles and manipulate paramagnetic air bubbles by magnetic repulsion^41–44^. A custom biocompatible ferrofluid was used to separate label-free cancer cells, extracellular vesicles, and HeLa cells from the blood^45–47^.

In recent years, more attention has been attracted to the isolation of sub-micro and nanoparticles, which is essential in several medical applications, especially those aimed at purification^48^. Lin et al. presented a two-elevation design, bottom and top channels, to enhance purification during separation^49^. Zeng et al. used an ultra-high magnetic field for the nanoparticle separation system^50,51^. A high-permeability material is constructed on the microchip and magnetized by two external magnets on one side of the channel to produce high-intensity gradients. They reported 95.0% purification of 0.2 μm particles from a mixture containing 1.0 μm particles; the purification increased to 98.2% by adding two magnets to the other side of the same microchannel^52^.

It can be observed that the sorting systems above are based on the axial incremental magnetic manipulation of the streamed entities to be separated. However, these systems have low throughput and a limited number of targets that can be sorted simultaneously^53^. One can use magnetophoretic sorting systems that utilize repulsion forces created by a magnetized ferromagnetic wire in a transversal magnetic arrangement to overcome such issues. The interaction between the magnetic microparticles and the magnetic gradients formed around a ferromagnetic wire was analyzed and used for different purposes^54–56^. Khashan introduced a novel microfluidic sorter that utilizes the repulsive magnetic force formed around an extended wire perpendicular to the external magnetic field and the flow direction^57,58^.

The behavior of the flowing magnetic beads inside a microfluidic channel with the described wire was experimentally demonstrated in invasive and non-invasive modes^59–62^. In the invasive mode, the magnetized element, such as the wire, is positioned within the sample flow. In contrast, the non-invasive mode involves embedding the magnetized element within the channel's walls or placing it in the vicinity of the wall without direct contact with the sample. In addition, sample focusing, the degree of separability of particles, and how they could be employed in applications such as filtration and purification were discussed. In another work, a numerical model was reported, and a sorting device based on a non-invasive wire was fabricated and tested^53^. The system's performance was demonstrated by simultaneously separating two and more magnetic beads.

**Figure 1:**
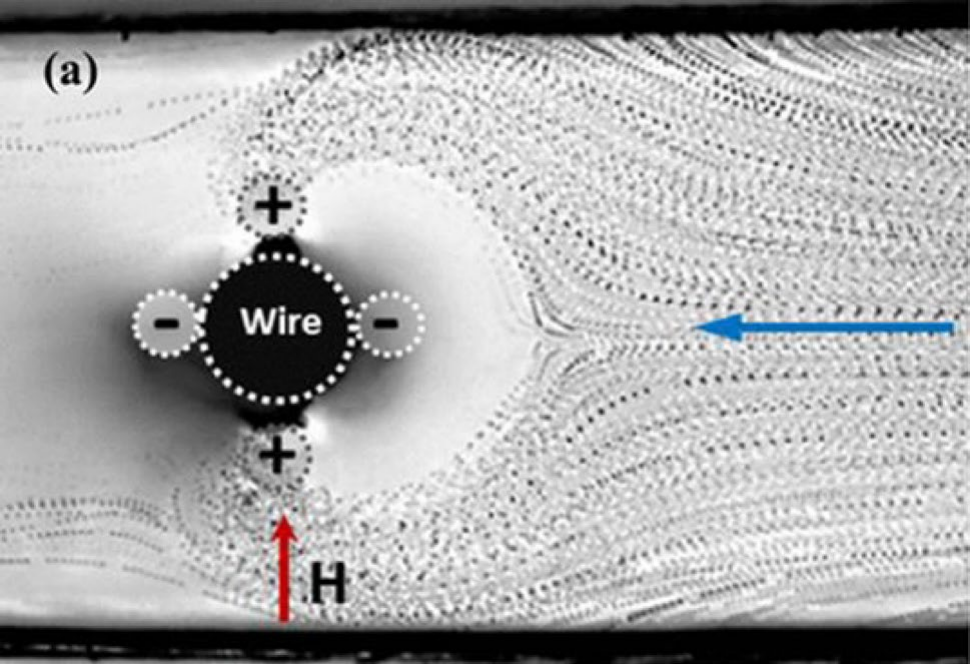
Bright-field microscopic images of magnetic particles under a transversal magnetic arrangement^53^.

Figure 1 shows a microscopic image of flowing magnetic particles under the effect of a transversal magnetic arrangement. The positive and negative signs indicate the sides of high and low magnetic field gradients, respectively. The external magnetic field *H* created by permanent magnets causes the ferromagnetic wire to form local repulsive and attractive regions around its angular span. As the injected magnetic beads approach the wire, they experience a repulsive effect emanating from the low magnetic gradient region. Consequently, they are pulled (or deflected) toward the attraction region (high magnetic field gradient). This principle can be utilized for multi-target sorting based on the distinct deflection toward different outlets. In addition, the throughput can be increased by scaling up the cross-sectional area of the channel in the transversal depth aligned with the length of the wire. This advantage can decrease the processing time and overcome the limitation of the throughputs associated with many existing techniques.

Based on a single non-invasive wire, we aim to simulate and compare two possible transversal magnetic configurations for a microfluidic separation system. These arrangements are utilized to sort different-sized magnetic particles based on a positive Magnetopherosis (pM) mode. Furthermore, the same systems are manipulated to activate a negative Magnetopherosis (nM) mode to separate label-free biological cells. The capabilities of the presented systems allow us to create a customized platform that can handle as many magnetic/nonmagnetic targets as possible simply by utilizing the device's simple and purchasable components tunability feature. Furthermore, the adopted microfluidic channel's separation chamber design is flexible when dealing with different particles' deflection trajectories based on their distinctive response. This advantage and the hydrodynamic focus make it possible to optimize the outlets' positions at the separation chamber to sort all injected entities completely. Moreover, the invasive wire distance from the sorting region is another critical parameter that controls the repulsive forces responsible for the separation process by its linear move. More importantly, a high throughput can be obtained without any drop in the separation performance by scaling up the depth of the channel and the extended wire.

This study aims to develop a highly efficient, high-throughput separation device by harnessing the combined effects of inertial and magnetophoretic forces. The research focuses on two magnetic arrangements: dual-magnet (DMA) and single-magnet (SMA). In the DMA setup, the wire is magnetized by positioning it between two magnets along its centerline, whereas the SMA utilizes a single magnet for magnetization.

The novelty of this work lies in designing negative and positive magnetophoresis-based separation devices that are highly tunable and capable of sorting numerous types of entities with high throughput. The study includes a comprehensive numerical analysis with experimental proof-of-concept using an in-house fabricated device. The SMA design was developed to overcome potential fabrication challenges encountered with the DMA. By using only one external magnet centered with the wire, the SMA allows for a larger microfluidic chip that is easier to prepare and handle. Additionally, the SMA enables an easily disposable chip with a custom-made stage. The lower magnetic forces affecting the wire in the SMA also simplify wire movement automation compared to the DMA. In addition, the SMA design provides more flexibility in designing the channel vents or outlets, as it has fewer spatial limitations than the DMA. Overall, this study enhances the development of a highly effective and fast separation device that can be adjusted to meet different needs and purposes.

## Theory

The governing equations are presented to model the movement of particles in a microfluidic channel under the influence of a magnetic field. The formulation will include the magnetic field, laminar flow, and the analysis of the forces acting on the particles.

### Magnetic field equations

The constitutive relation that relates the magnetic flux density $\overrightarrow{B}$ [T] and the external magnetic field of a strength $\overrightarrow{H}$ [A/m] is given by:

1 $$\overrightarrow{B} = {\mu }_{o} \left(1 + \chi \right) \overrightarrow{H}$$

where $\chi$ is the volumetric magnetic susceptibility and ${\mu }_{o}$ ($4\pi \times {10}^{-7}$ H/m) is the magnetic permeability of free space. Since a permanent magnet provides the magnetic field, Maxwell's equations are reduced to^63^:

2 $$\nabla .\overrightarrow{B}=0$$

3 $$\nabla \times \overrightarrow{H}=0$$

Equation (3) can be rewritten in terms of a magnetic scalar potential $\phi$ as^64^:

4 $$\overrightarrow{H}=-\nabla \phi $$

For a circular ferromagnetic wire centered at (0,0), with a radius $a$ [m] and exposed to a uniform one-dimensional external magnetic field, ${\overrightarrow{\text{H}}}_{o}={H}_{o}{e}_{y}$, $\phi$ can be expressed as follows^7^:

5 $$ \phi = - H_{o} y + k_{p} H_{o} a^{2} \frac{y}{{\left( {x^{2} + y^{2} } \right)}},\;\;r = \sqrt {x^{2} + y^{2} } { } > a $$

6 $$ k_{p} = \frac{{\mu_{w} - \mu_{o} }}{{\mu_{w} + \mu_{o} }}{ }\;\;{\text{(for}}\;\mu_{o} \cong \mu_{f} ) $$

where ${\mu }_{w}$ and ${\mu }_{f}$ are the magnetic permeabilities for the wire and surrounding fluid, respectively, and $r$ is the cylindrical coordinate of the distance. Combining (5) and (6) and substituting in (4)^53^:

7 $$\overrightarrow{H}=-\nabla \phi =\frac{{H}_{o}}{{\left({x}^{2}+{y}^{2}\right)}^{2}}[\left(2{a}^{2}kxy\right){e}_{x}+\left({\left({x}^{2}+{y}^{2}\right)}^{2}-{a}^{2}k\left({x}^{2}-{y}^{2}\right)\right){e}_{y}]$$

### Fluid flow equations

The continuity and Navier–Stokes equations govern the fluid flow field. For an incompressible and Newtonian fluid, the equations can be written as follows^65^:

8 $$\nabla .\overrightarrow{u}=0$$

9 $$\frac{\partial \overrightarrow{u}}{\partial t}+\overrightarrow{u}.\nabla \overrightarrow{u}=-\frac{\nabla P}{\rho }+g+\nu {\nabla }^{2}\overrightarrow{u}$$

where $\overrightarrow{u}$ [m/s] is the fluid velocity, $P$ [Pa] is the fluid pressure, $\nu$ [m^2^/s] is the fluid kinematic viscosity, $g$ is the acceleration of gravity and $\rho$ [kg /m^3^] is the fluid density.

### Particle transport equation

By considering the forces acting on a particle moving in the microfluidic channel, the transport of the particles will be expressed using Newton's second law as follows^66^:

10 $${m}_{p}\frac{d{\overrightarrow{u}}_{p}}{dt}={\overrightarrow{F}}_{m}+{\overrightarrow{F}}_{d}+{\overrightarrow{F}}_{g}+{\overrightarrow{F}}_{b}+{\overrightarrow{F}}_{l}$$

where ${m}_{p}$ [kg] is the mass of the particle, ${\overrightarrow{u}}_{p}$ [m/s] is the particle's velocity, ${\overrightarrow{F}}_{m}$, ${\overrightarrow{F}}_{d}$, ${\overrightarrow{F}}_{g}$, ${\overrightarrow{F}}_{b}$, and ${\overrightarrow{F}}_{l}$ are the magnetic, drag, gravitational, buoyancy, and lift forces, respectively, where force is in newtons [N]. The considerable forces are ${\overrightarrow{F}}_{m}$ and ${\overrightarrow{F}}_{d}$. The remaining forces are relatively less significant and can be neglected^53,66,67^. The magnetic force can be described as follows^53^:

11 $${\overrightarrow{F}}_{m} =\frac{1}{2}{\mu }_{o}({\chi }_{p}-{\chi }_{f}){V}_{p}\nabla {H}^{2}$$

where ${\chi }_{p}$ and ${\chi }_{f}$ are the susceptibilities of the particle and carrying fluid, respectively, and ${V}_{p}$ [m^3^] is the volume of the particle. Considering the magnetic field around the magnetic wire (Eq. 7), the magnetic force components can be obtained as follows^53^:

12 $${F}_{mx}=-2{\mu }_{o}\left({\chi }_{p}-{\chi }_{f}\right){V}_{p}{H}_{o}^{2}{a}^{2}k\frac{\left(k{a}^{2}-{x}^{2}+3{y}^{2}\right)x}{{\left({x}^{2}+{y}^{2}\right)}^{3}}$$

13 $${F}_{my}=-2{\mu }_{o}\left({\chi }_{p}-{\chi }_{f}\right){V}_{p}{H}_{o}^{2}{a}^{2}k\frac{\left(k{a}^{2}-{3x}^{2}+{y}^{2}\right)y}{{\left({x}^{2}+{y}^{2}\right)}^{3}}$$

where $k$ is defined based on the saturation magnetization of the wire ${M}_{ws}$ as follows^53^:

14 $$ k = \left[ {\begin{array}{*{20}l} {1.0} \hfill & {if \;H_{o} \le \frac{{M_{ws} }}{2}; not - saturated} \hfill \\ {\frac{{M_{ws} }}{{2H_{o} }}} \hfill & {if \;H_{o} > \frac{{M_{ws} }}{2}; saturated} \hfill \\ \end{array} } \right] $$

The drag forces can be described as:

15 $${\overrightarrow{F}}_{d}=6\pi \eta {r}_{p}(u-{u}_{p})$$

where $\eta$ [Pa s] is the viscosity of the fluid and ${r}_{p}$ [m] is the radius of the particle. Lastly, the particle's trajectory position ${\overrightarrow{x}}_{p}(t)$ is presented as^53^,

16 $$\frac{d{\overrightarrow{x}}_{p}}{dt}={\overrightarrow{u}}_{p}$$

## Numerical simulations

**Figure 2:**
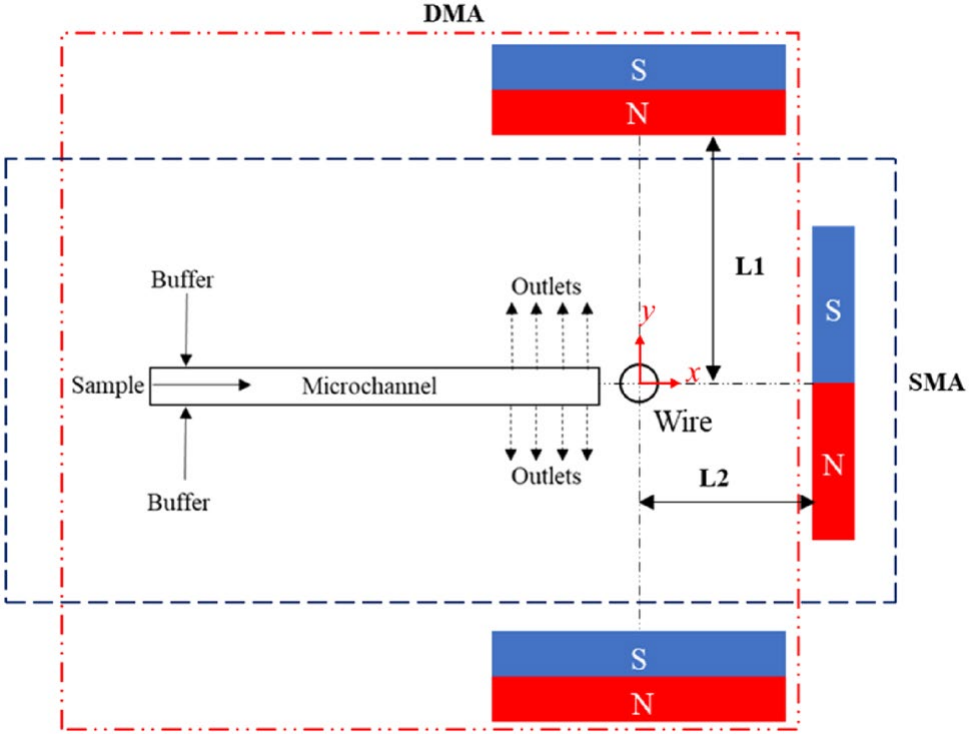
Schematic diagram for the dual-magnet arrangement (DMA) and the single-magnet arrangement (SMA) represented by the red and blue dashed lines, respectively.

Figure 2 shows a schematic diagram of the magnetic arrangements for the proposed systems. The systems' main components are the permanent magnet(s), the ferromagnetic wire, and the microfluidic channel. The DMA illustrates the magnetization of the wire by positioning it in the centerline of two magnets, while in the SMA, the magnetization is created by a single magnet. Therefore, the studies are carried out for two modes for each magnetic configuration: the pM-based DMA (DMA-pM), the pM-based SMA (SMA-pM), the nM-based DMA (DMA-nM), and the nM-based SMA (SMA-nM). The adopted coordinate system for both simulations and experiments is illustrated in Fig. 2, with the origin centered on the wire.

### Computational domains

**Figure 3:**
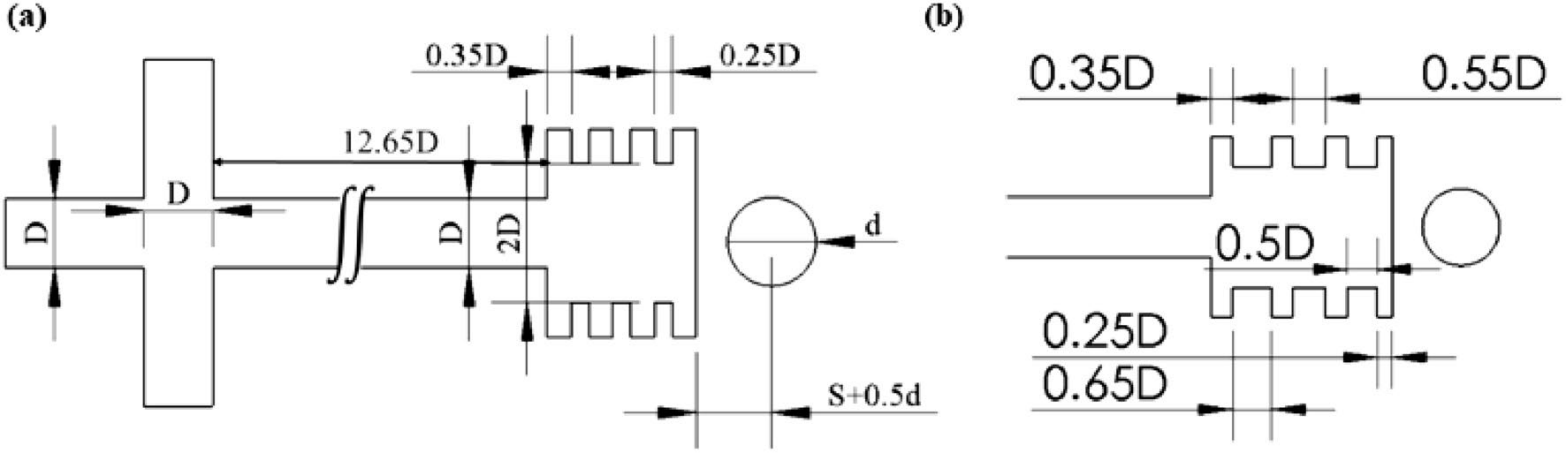
Microchannels' dimensions, not to scale, used in each system; (**a**) Design 1 is used in the SMA-pM and DMA-nM, (**b**) design 2 is used in the DMA-pM and SMA-nM. The inlet section and the main channel length are the same as in Design 1.

Figure 3 shows the microchannel and ferromagnetic wire domains used in the simulation of each system with dimensions with respect to the main channel width (D = 200) [μm]. The microchannels' geometry is based on a published design by Khashan with slight adjustments^57,58^. The center of the 254 [μm] diameter (d) wire is set as the coordinate origin for the simulation models. The microfluidic channel is positioned along the x-axis to ensure a symmetrical effect during separation. Two lateral buffer streams hydrodynamically focus the sample flow, and all inlets are equal in width. The eight outlets' positions in the separation chamber are designed to correspond with the deflected trajectories of the magnetic targets induced by the repulsive forces according to their distinctive magnetic responses. The distance (S) between the wire and the closed end of the separation chamber is kept at 100 [μm] (edge-to-edge) in both microchannels. Still, the outlets' positions and dimensions are slightly different due to the magnetic characteristics of each arrangement. In the DMA, the wire is magnetized by two 12 × 3 [mm^2^] magnets with magnetization through the 3 [mm] dimension; the distance between the edge of each magnet to the wire center is (L1 = 3000) [μm].

In the SMA, the wire is magnetized by a 12.7 × 3.175 [mm^2^] magnet, magnetization through the 12.7 [mm] dimension. The magnet is positioned at a distance (L2 = 530 [μm]) to the right of the wire's center. Finally, the air domain is 60 × 60 [mm^2^]. In the nM mode, magnets' domains are flipped while keeping all other parameters the same.

### Materials and properties

The wire is assumed to be a Permalloy (ESPI Metals, USA), and grade N52 permanent magnets (remanent flux density = 1.48 [T]) are considered for the external magnetic field. The specifications of a disc magnet (Neomagnete, Germany) and a block magnet (B428-N52, K&J Magnetics, USA) are considered for the DMA and SMA, respectively. Superparamagnetic beads M-450, M-280, and MyOne are used along with nonmagnetic beads as the injected microparticles in the pM mode systems. Red blood cells (RBCs), white blood cells (WBCs), and the Prostate cancer cell line (PC3-9) are considered to verify the functionality of the nM mode arrangements. The 0.05 × EMG 408 (Ferrotec, USA) is adopted as the ferrofluid medium because of its reasonable biocompatibility and optical properties^36^. All properties of the materials used in simulations are summarized in Table 1^27,68–73^.

**Table 1:** Material properties used in simulations.

		$\rho$^a^	$r$^b^	$\chi$^c^	$\eta$^d^
Wire		–	127	90,000	
pM mode	Medium	1000	–	0	0.001
	Nonmagnetic	1800	1	0	–
	MyOne	1791	1.05	1.43	–
	M-280	1538	2.8	0.923	–
	M-450	1578	4.5	1.58	–
nM mode	Medium	1070	–	0.025	0.00105
	RBCs	1100	4	− 3.69 × 10–6	–
	WBCs	1080	7	− 9.9 × 10–6	–
	PC3-9	1070	9.75	− 9.5 × 10–6	–

^a^$\rho$ is the density [kg/m^3^].

^b^$r$ is the radius [μm].

^c^$\chi$ is the magnetic susceptibility.

^d^$\eta$ is the viscosity [Pa s].

### Physics and boundary conditions

The simulation takes the magnetic fields and hydrodynamics and couples them to examine the effect of the magnetophoretic and drag forces on the microparticles of varied sizes and properties using the COMSOL Multiphysics package. The external magnetic field generated by the permanent magnets is set in the y-direction and x-direction in the pM and nM modes, respectively. In the laminar flow module, all the inlets' velocities are considered fully developed flows, and a zero static pressure boundary condition at the outlets is applied to consider a free flow. The sample inlet velocity (*V*_*s*_) and buffer inlet velocity (*V*_*b*_) values for each system are summarized in Table 2. The analysis of the magnetophoretic and drag forces experienced by the injected particles and their transport is carried out by the particle tracing module. Finally, the magnetic and flow fields are solved in a stationary study; then, the obtained solution is coupled with a time-dependent study to solve the particles' trajectories in the microfluidic channel.

**Table 2:** Inlet velocity boundary conditions used in simulations.

	${V}_{s}$^a^	${V}_{b}$^b^
DMA-pM	0.5	5
SMA-pM	0.5	2
DMA-nM	7	9
SMA-nM	20	33

^a^${V}_{s}$ is the sample inlet velocity [mm /s].

^b^${V}_{b}$ is the buffer inlet velocity [mm/s].

## Experimental methods

This section presents detailed experiments conducted to investigate the effects of magnetic fields on particles. It also examines the use of magnetic fields for particle switching, sorting, and separation. Additionally, it demonstrates the device's pM-based sorting capability by sorting a sample containing two magnetic and one nonmagnetic particle. The fabrication methodology of the microchannel, the materials selected for this purpose, and the experimental setup are clarified, with a particular emphasis on the orientation of the magnets. The magnets are arranged according to the DMA, adhering to the parameters of Geometry Design in Fig. 1. This approach validates the theoretical models and explores the practical implications and applicability of the findings within a controlled experimental framework.

### Microfabrication

**Figure 4:**
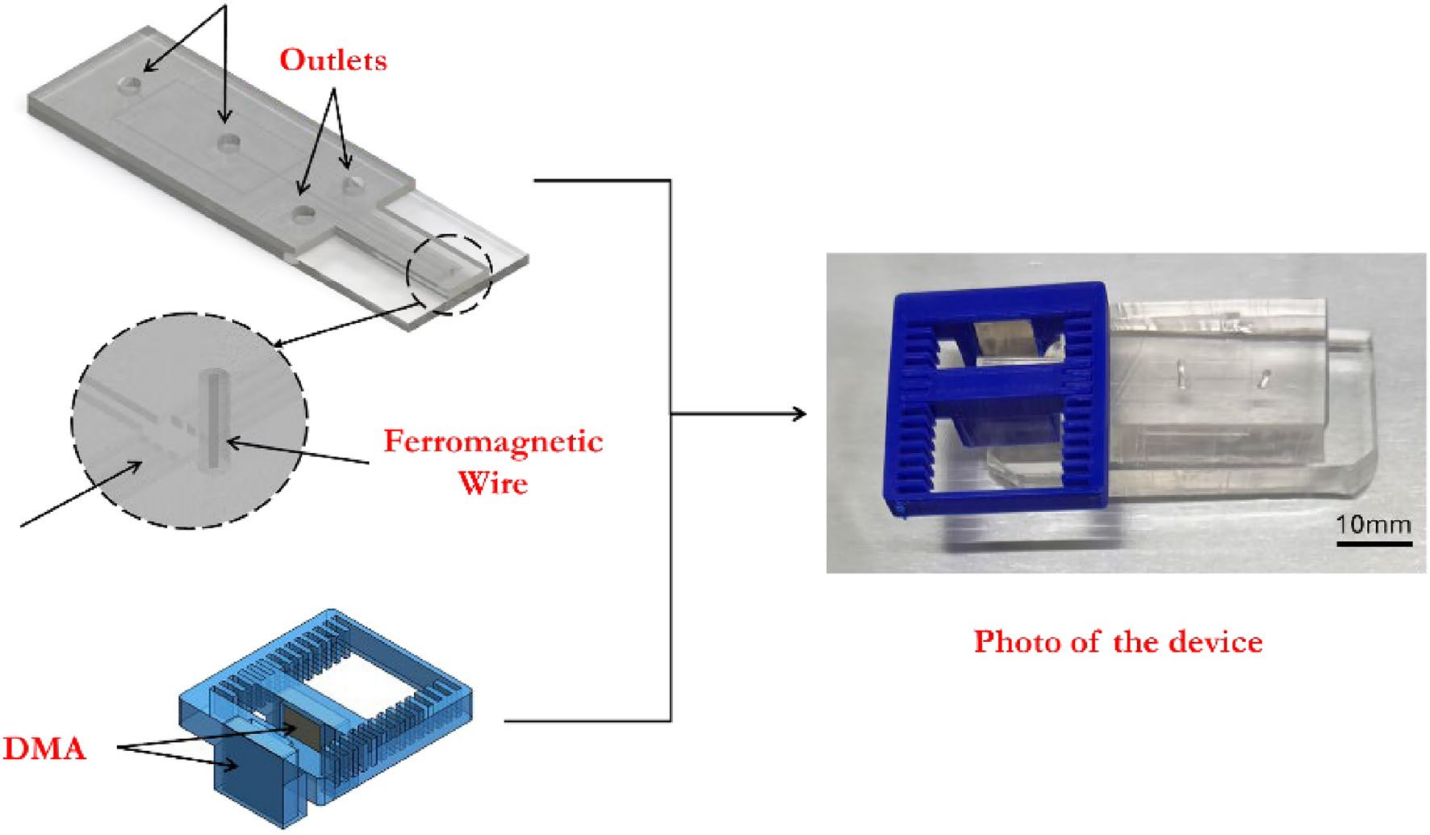
Schematic diagrams of the fabricated microfluidic device and the magnet holders, along with a photograph of the completed device.

The proposed microdevice comprises a microchannel with two inlets for the buffer, a single inlet for particle entry, several outlets, ferromagnetic wire, and magnetic holders with a permanent magnet, as depicted in Fig. 4. The selected design for particle sorting is shown in Fig. 4. The fabrication process involves two main steps. Firstly, the microchannel is created using the soft lithography technique, while the magnetic holder is made using a 3D printing approach (UltiMaker S5 3D printer). Blue polylactic acid (PLA) is used as the printing material. The holder is designed with a special feature that allows for adjusting the distance between two magnets by 1 mm increments.

**Figure 5:**
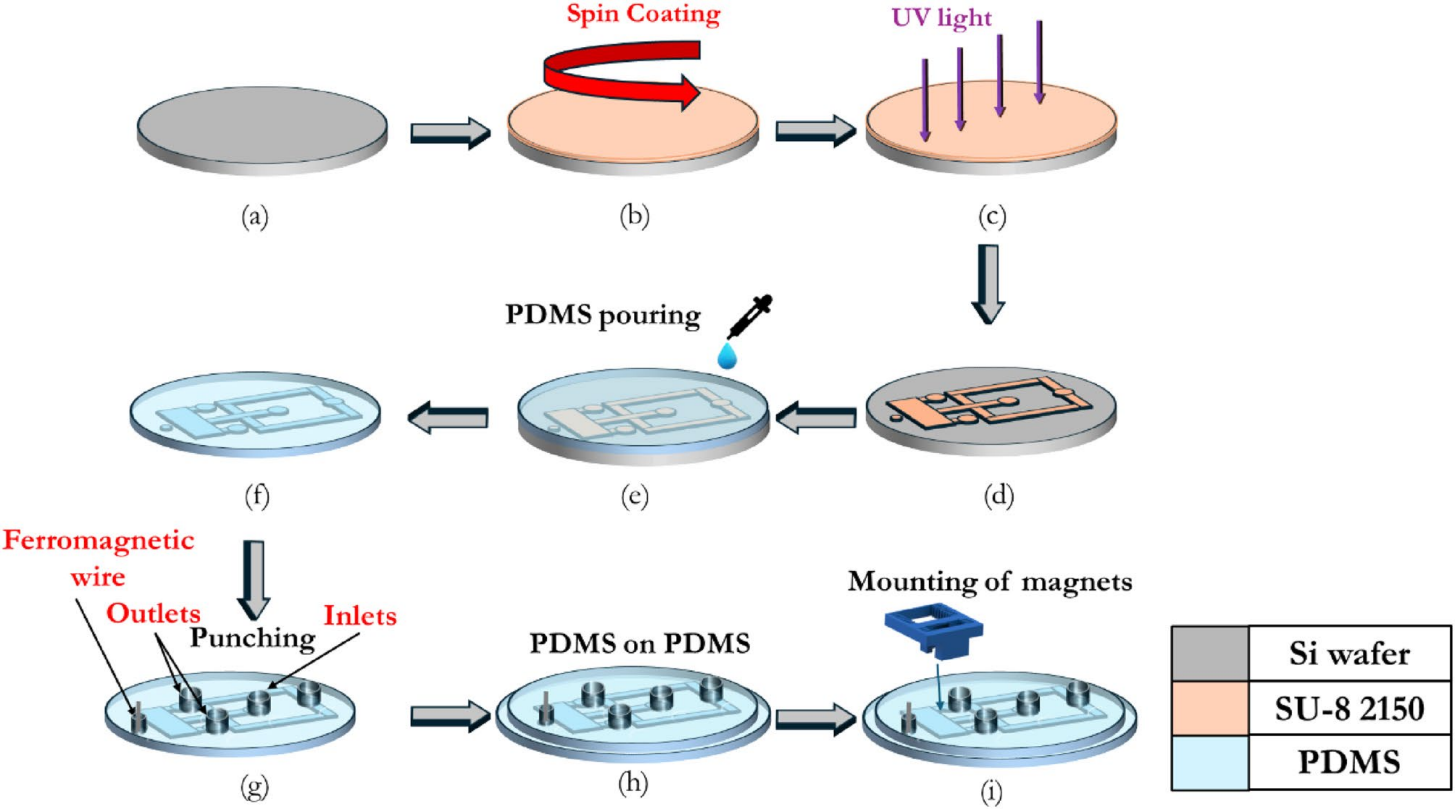
Schematic diagram for the microchannel fabrication process and the magnetic wire insertion.

The microchannel component of the device is fabricated using the standard soft lithography method, as depicted in Fig. 5. Polydimethylsiloxane (PDMS) is selected as the material for both the microchannel and its base. A 100 µm height mold for the PDMS is created using SU-8 2075 and patterned using a direct lithography system (Kloe Dilase 650). The ferromagnetic wire is precisely inserted into the PDMS alongside the creation of the holes, as illustrated in Fig. 5g. Finally, the microchannel and the base are bonded using oxygen plasma treatment on both surfaces. The magnet holder is then mounted onto the channel, making the device operational.

### Experimental setup

The pM-based device is tested using a DI buffer and several samples with different particles (Microparticles GmbH, Germany), including polystyrene particles with iron oxide content, PS-MAG beads with sizes of 10.43 µm (PS-MAG-S2874) and 5.61 µm (PS-MAG-S1684) and nonmagnetic polystyrene beads of 18.46 µm (RS/Q-R-L2619), suspended in a DI medium. The flow of the buffer and the samples is controlled by a syringe pump, maintaining a buffer-to-inlet flow ratio of 10:1. A Reynolds number (Re) of 3 is selected for sorting the particles. Subsequently, the movement and behavior of the particles are observed using a high-speed camera attached to a microscope.

## Results and discussion

### Experimental results

The experimental section consists of three distinct experiments. Firstly, we examine the effect of the magnetic field on switching particles of the same size between the outlets. Secondly, we study the influence of the magnetic field on sorting particles of various sizes. Lastly, we investigate the effect of this device on separating three types of particles (two magnetic and one non-magnet).

#### Switching microparticles of the same size between the outlets

**Figure 6:**
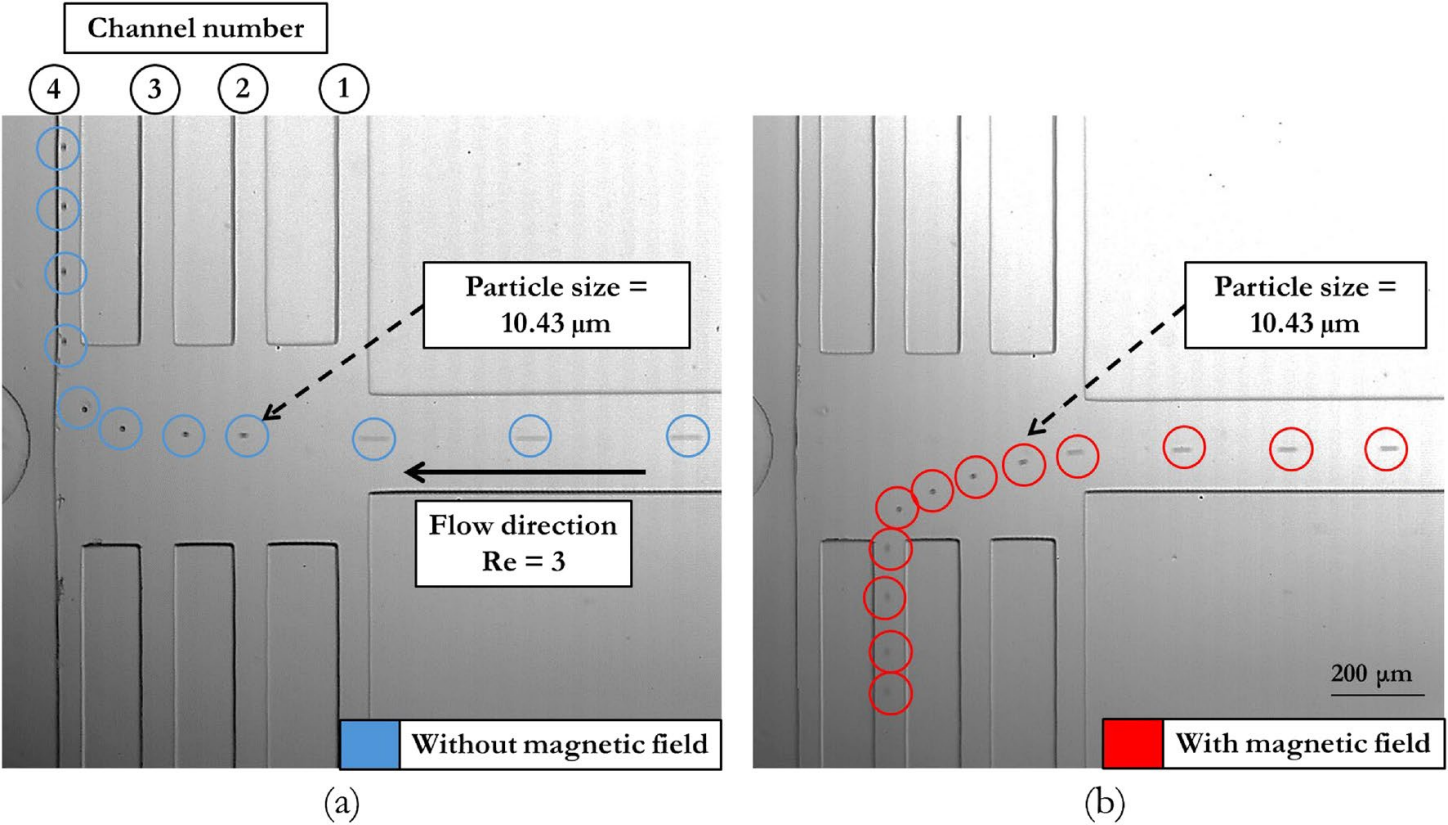
Experimental results showing the trajectory of 10.43 µm particles. (**a**) without magnetic field and (**b**) with magnetic field under DMA. The experiment used two N52 nickel-plated magnets with size 0.5 × 0.5 × 0.125 inches.

Initially, particles of a single size, 10.43 µm, are examined to observe the influence of the magnetic field on their behavior. In Fig. 6a, it is shown that the particle exits via the 4th channel in the absence of a magnetic field. This is due to the hydrodynamic force pushing them toward the last outlet. However, with the application of the magnetic field, a shift of the particle towards the 3rd channel is observed, as shown in Fig. 6b. The deviation of the particle trajectory toward the third outlet results from the balance between the magnetic and hydrodynamic forces. Given the symmetric design of the channel, the direction of the particle's movement, either upwards or downwards, is determined by its entry position.

#### Sorting particles of different sizes

**Figure 7:**
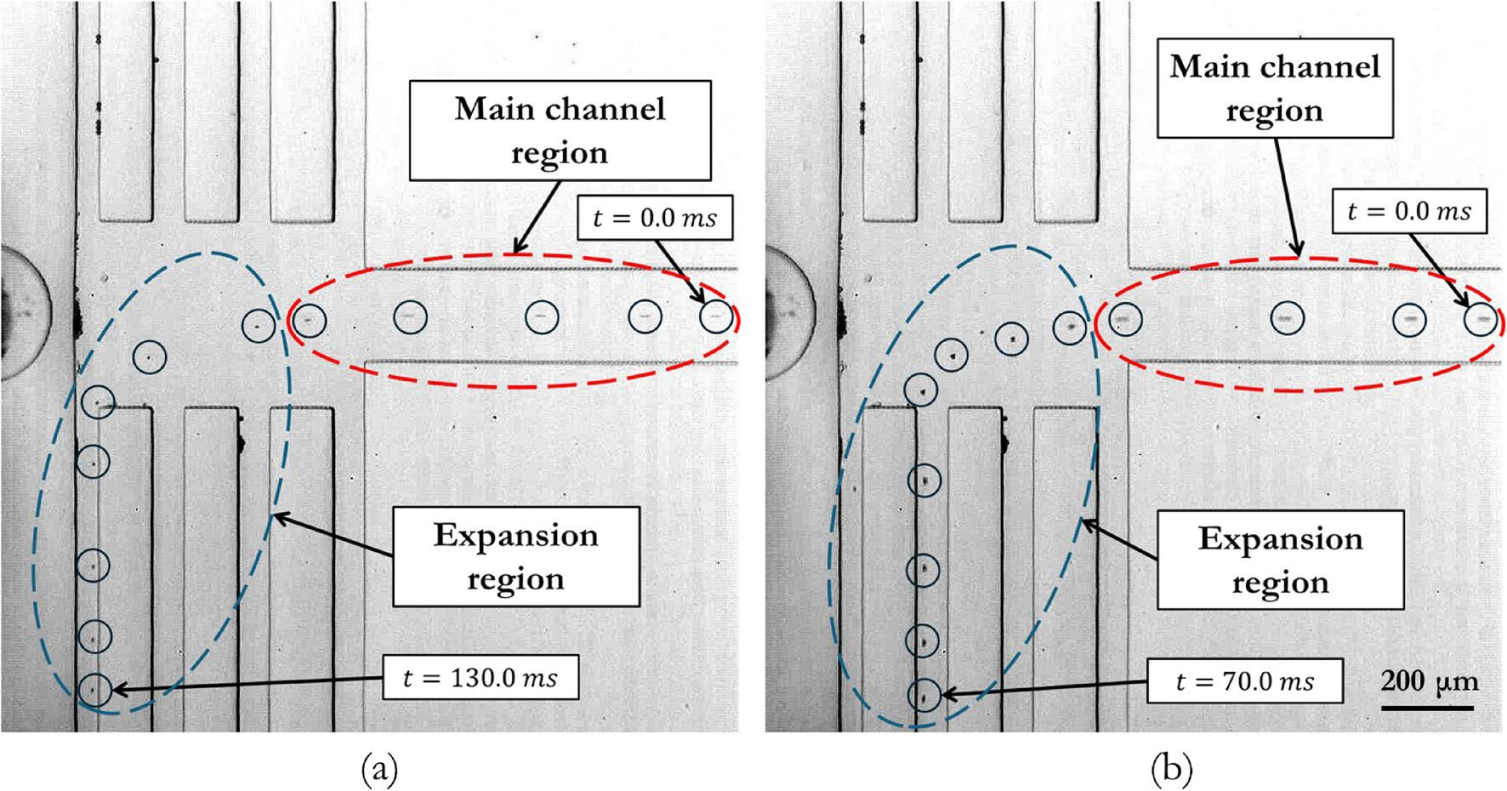
Particle Trajectories for Sorting Under Dual-Magnet Arrangement (DMA) with Magnetic Field. (**a**) Trajectory of 5.61 µm particles exiting the 4th channel. (**b**) Trajectory of 10.43 µm particles exiting the 3rd channel. The experimental setup includes two N52 Nickel Plated magnets sized 0.5 × 0.5 × 0.125 inch.

In the experimental results shown in Fig. 7, efficient sorting of different-sized particles is achieved by leveraging the differences in magnetic and hydrodynamic forces among particles of various sizes. In the main channel region, the magnetophoretic force is negligible, and particles of all sizes are solely influenced by fluidic drag. However, as they transition into an expansion region where magnetic resistance becomes significant, a noticeable change in trajectory occurs due to the introduction of magnetic forces opposing the movement of these particles. Larger particles (10.43 µm) experience greater magnetic forces, causing them to deviate more swiftly from the magnetic field than smaller particles (5.61 µm). The discrepancy in the movement of these particles from regions of high velocity to regions of low velocity validates the device's ability to separate particles according to their size, which is influenced by magnetic content and hydrodynamic characteristics.

#### Separation of magnetic and nonmagnetic particles

**Figure 8:**
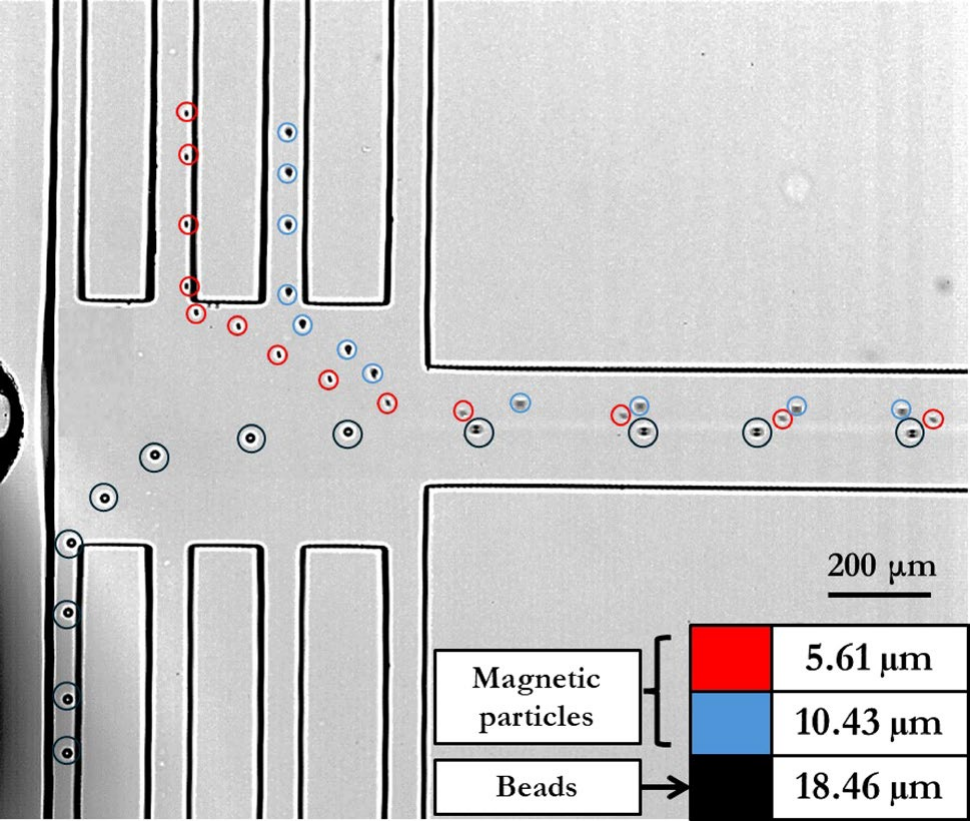
The experimental results illustrate the separation process under DMA. The trajectories of three types of particles, 5.61 µm and 10.43 µm magnetic particles and 18.46 µm nonmagnetic particles, are shown. The experimental setup includes two N52 nickel-plated magnets sized 0.5 × 0.5 × 1 inch.

In this part of the study, we explore the influence of the magnetic field on the trajectory of three different particles, as depicted in Fig. 8. The Magnetophoretic force (FMEP) is directly proportional to the square of the particle's radius as per Eqs. 12, and 13. Consequently, particles with a larger radius, indicated by blue circles, tend to move towards the second channel, while those with a smaller radius, marked in red, shift towards the third channel. Despite having the largest diameter of 18.46 µm, the nonmagnetic beads are not influenced by the magnetic force and proceed to the fourth channel, nearest to the wire, due to the high hydrodynamic force acting on them. Thus, the capability of this device to separate particles using the design in Fig. 1 by the Dual-Magnet Arrangement (DMA) orientation is demonstrated.

### Simulation verification and mesh independence

**Figure 9:**
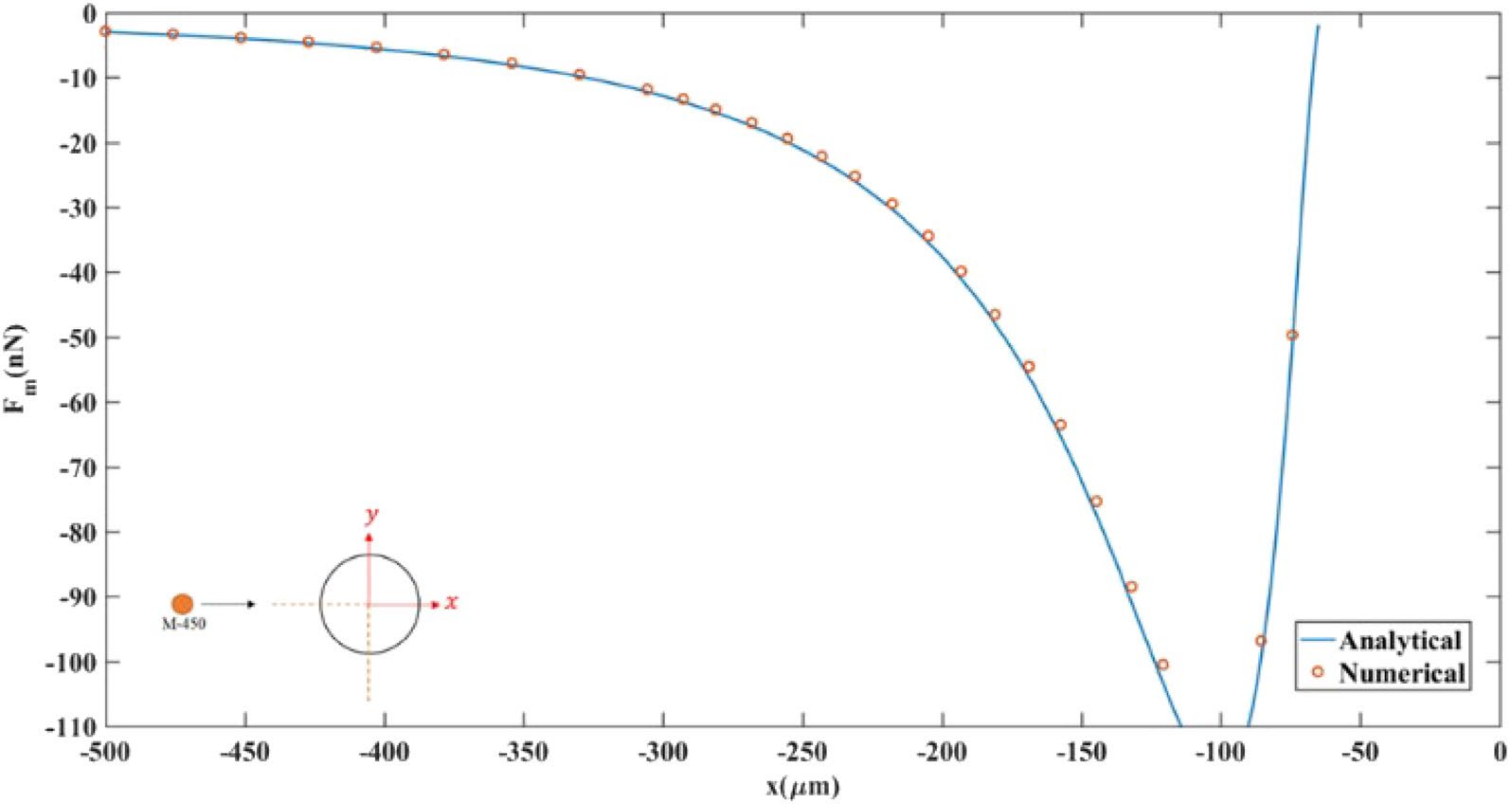
Magnetophoretic forces acting on an M-450 bead moving along the longitudinal direction of the wire vicinity and y = 0. The coordinate system is illustrated in Fig. 2.

The numerical solution of the magnetophoretic force (F_m_) acting on an M-450 particle was calculated to validate the simulation results. The medium fluid was nonmagnetic, and a 127 [μm] diameter wire was centered at a 1 × 1 [mm^2^] domain and exposed to a magnetic flux density of 1.48 [T] in the y-direction. The analytical values were calculated by solving Eqs. (7, 12, 13), while the magnetic field distribution for the computational domain was obtained after solving it time independently, then the force components were calculated. The results in Fig. 9 show a good agreement between the analytical and numerical solutions. Also, the maximum deviation was 4.5295%, and the average error was only 3.1341%.

**Figure 10.**
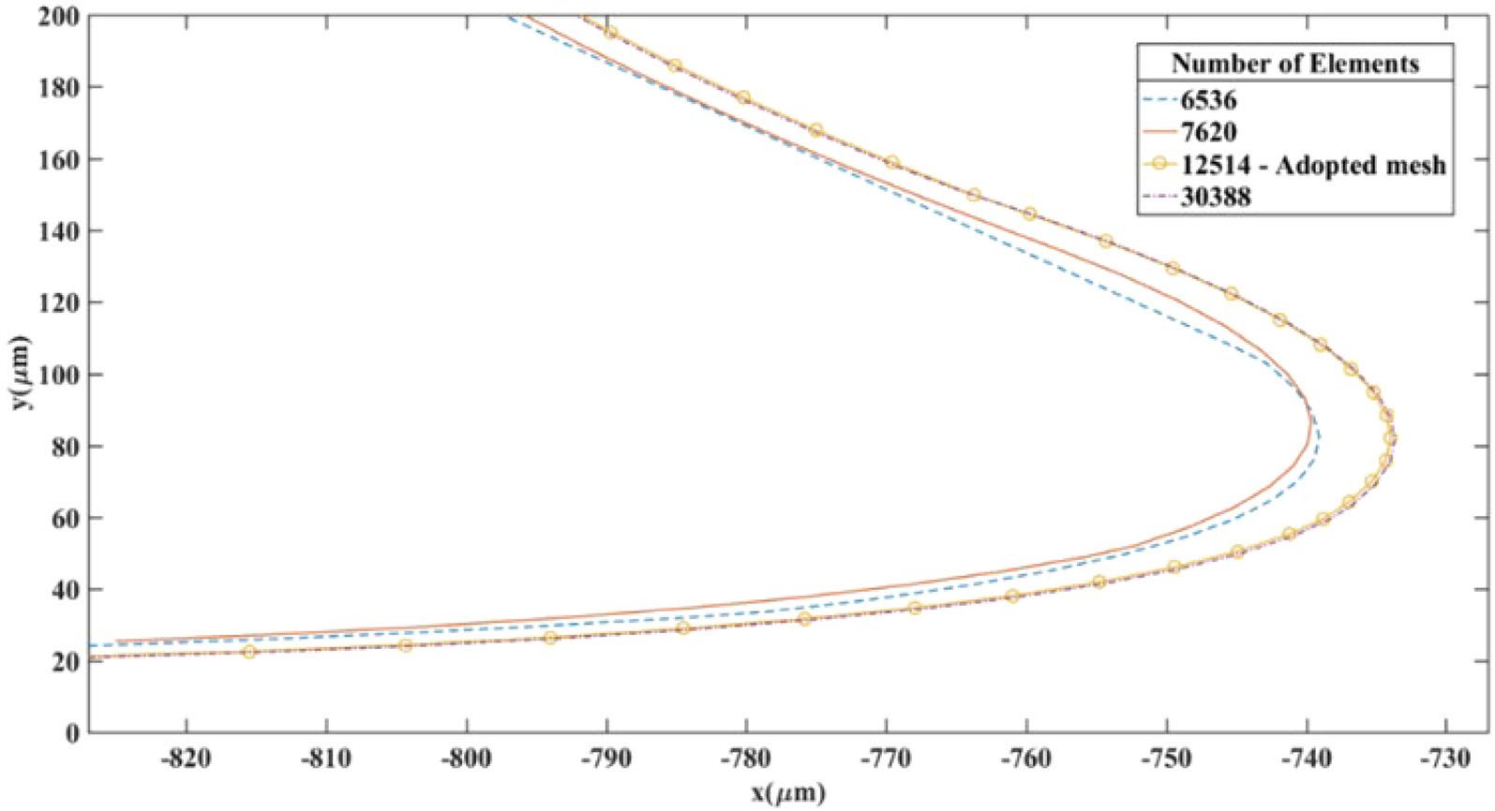
Plot showing the trajectories of M-450 particles at various mesh sizes as part of a mesh dependency study. The coordinate system is illustrated in Fig. 2.

The mesh size used in the models was examined for solution dependency. For this test, the M-450 trajectory in the separation chamber for the DMA-pM case was plotted using four different mesh sizes, as shown in Fig. 10. The trajectory was almost identical using finer meshes with 12,514 and 30,388 elements, respectively. Therefore, we adopted 12,514 elements throughout the study to balance the computational cost and accuracy.

### Magnetic characteristics

#### Positive magnetophoresis mode

**Figure 11:**
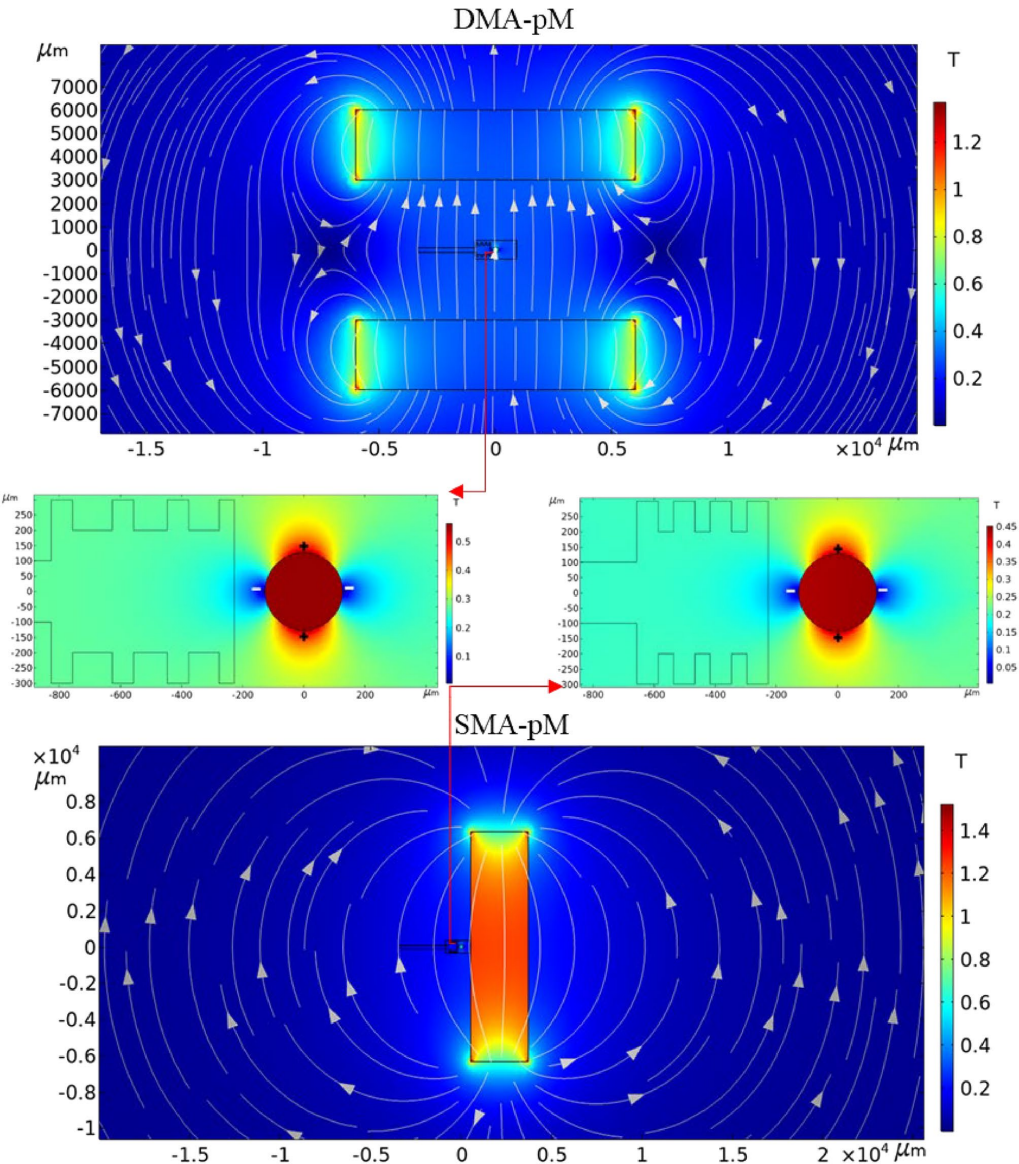
Magnetic flux density surface plot and magnetic field vectors with enlarged views on the wire for the pM arrangements.

The direction of the magnetic field lines and the magnetic flux density distribution for each arrangement are depicted in Fig. 11. The figure illustrates the magnetic field in the y-direction by the permanent magnets and the symmetrical magnetization of the ferromagnetic wire. The magnetic flux density induced by the wire, in each case, is illustrated in an enlarged view where it can be observed that the wire allows the magnetic field gradients to be locally amplified around its angular span, thus giving rise to the magnetic force on the particles entering the separation region. The positive and negative signs indicate the sides of high and low magnetic field gradients, respectively.

**Figure 12:**
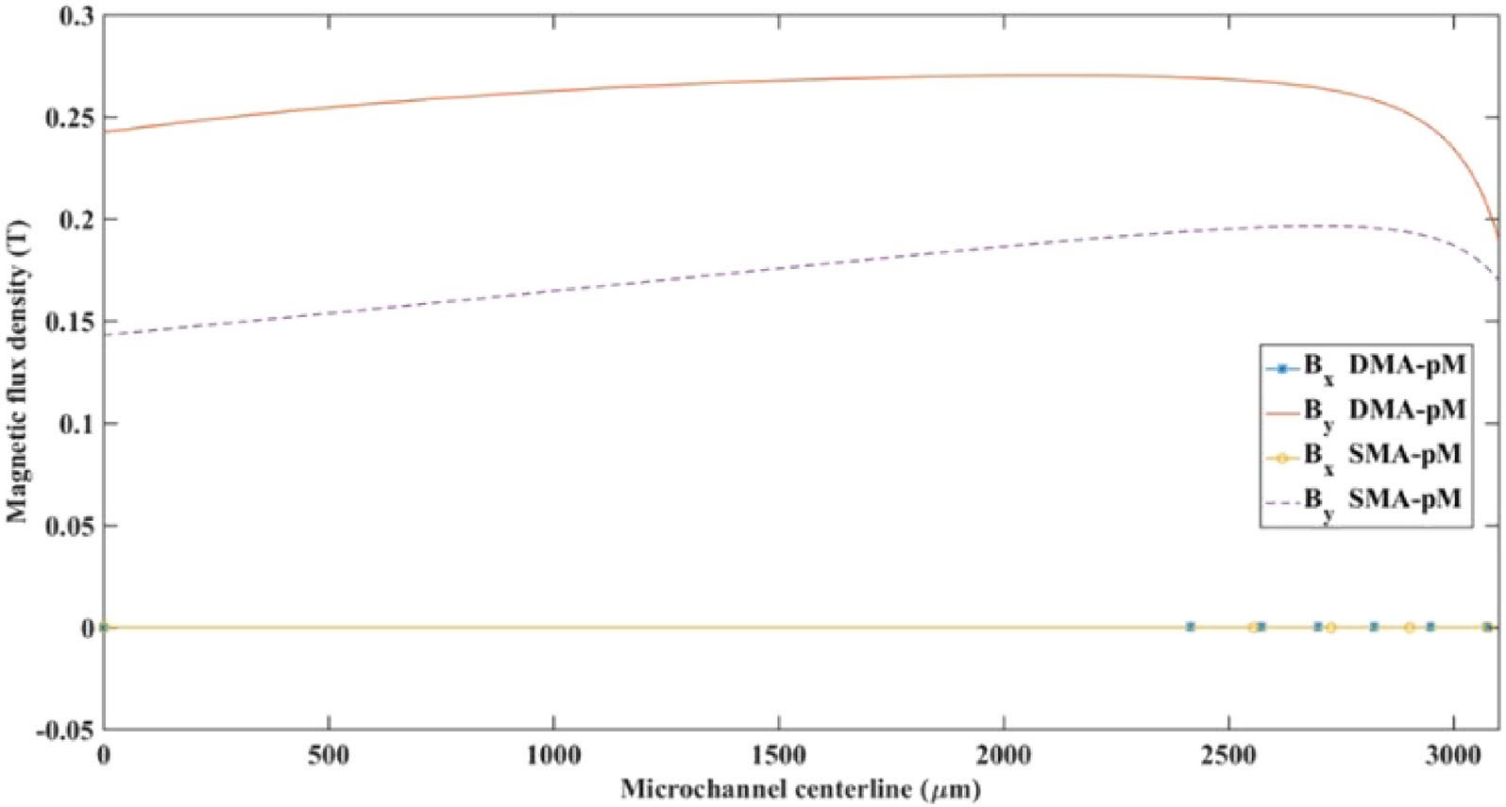
Magnetic flux density components, *B*_x_ and *B*_y_, along the channel’s axial centerline (*y* = 0) for the two magnetic arrangements in pM systems.

Figure 12 shows the computed magnetic flux density components *B*_*x*_ and *B*_*y*_ along the channel's centerline length. While *B*_*x*_ is almost zero, *B*_*y*_ distribution far from the separation region, it does not experience steep changes along the channel centerline. The magnitude of the magnetic field flux undergoes sharp changes as it approaches the wire's vicinity in the x-direction due to the local repulsive regions, which correspond to negative gradients.

**Figure 13:**
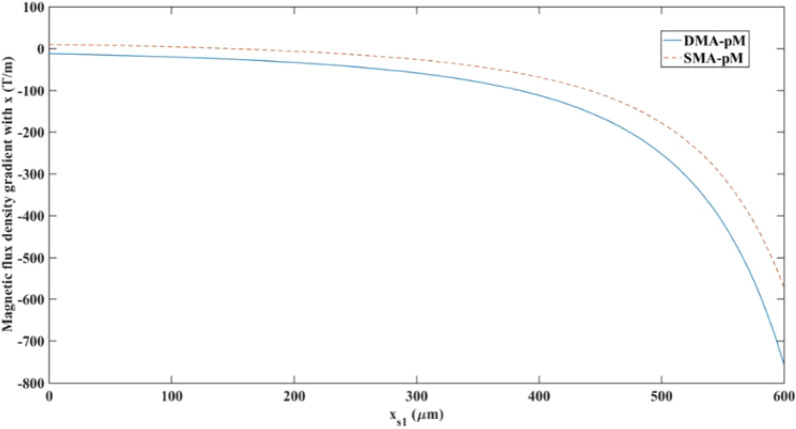
Magnetic flux density gradient in the x-direction along cutline x_s1_ and y = 0 [μm] in the separation region for the pM systems.

The magnetic gradient in the x-direction was explored in the separation chamber by computing it along a 600 [μm] length cutline (x_s1_) at y = 0 [μm], as presented in Fig. 13. The x-axis marks “0” as the beginning of the separation chamber and “600” as the end of the chamber's wall near the wire. The DMA-pM showed higher gradients than the SMA-pM because of the larger magnetic intensity created by the two magnets. However, in both cases, the gradient increased as it approached the edge of the channel near the wire, while before the separation region, it reached low values. The region before the separation chamber has been excluded from this plot, as its variation was found to be negligible.

#### Negative magnetophoresis mode

**Figure 14:**
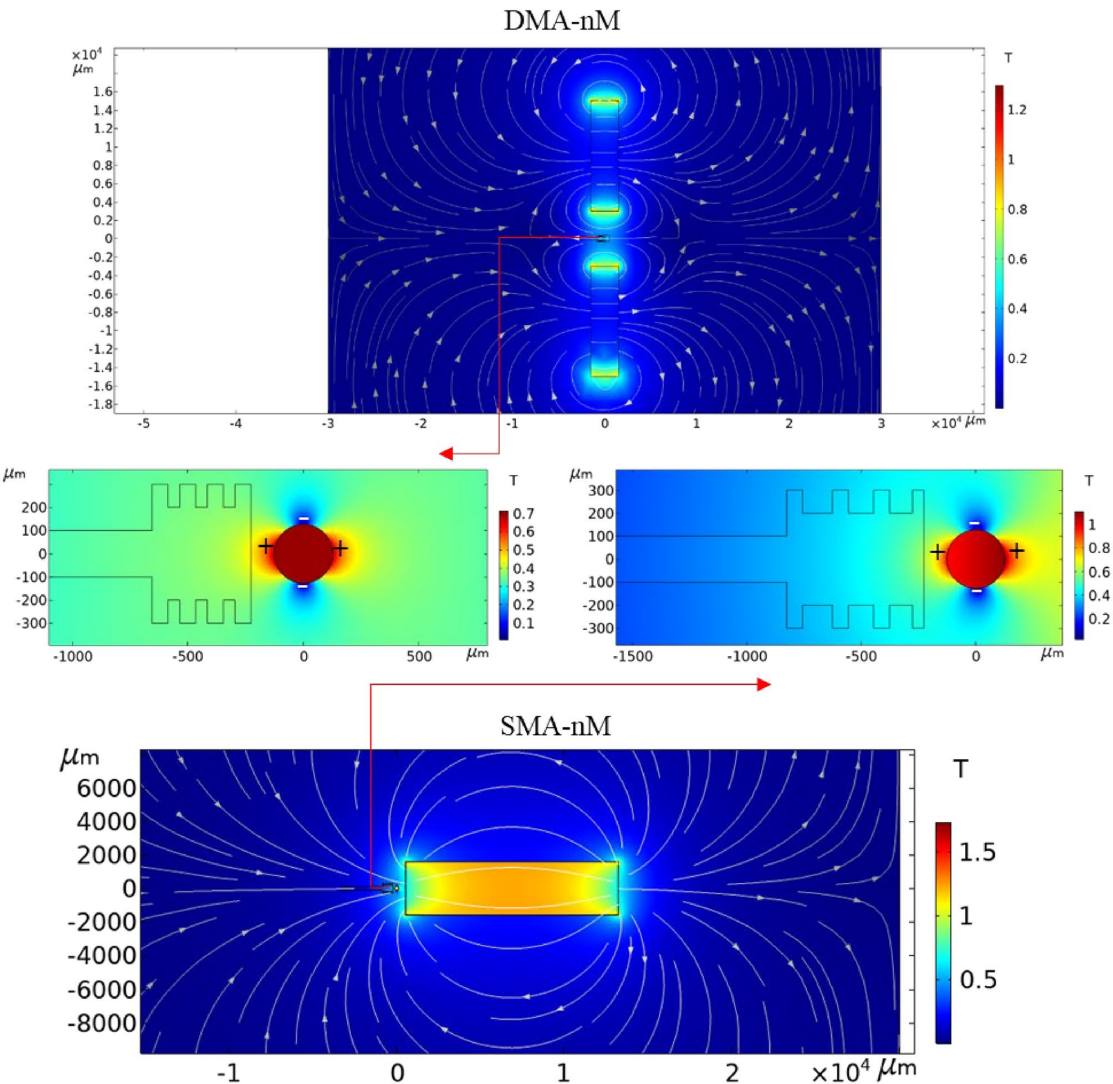
Magnetic flux density surface plot and magnetic field vectors with enlarged views on the wire for the nM arrangements.

**Figure 15:**
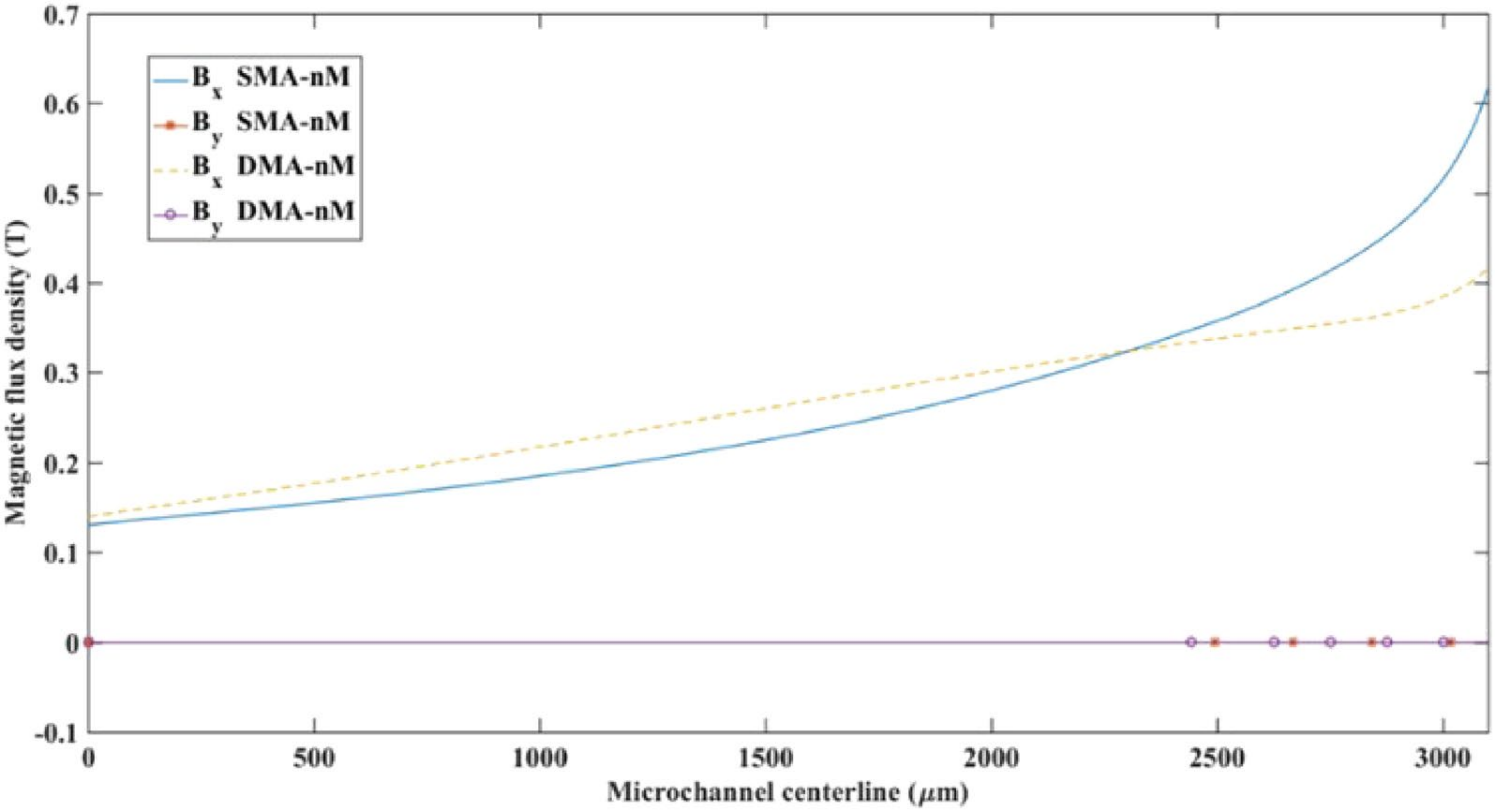
Magnetic flux density components *B*_x_ and *B*_y_ along the microchannel's centerline (y = 0) for the two magnetic arrangements in nM systems.

**Figure 16:**
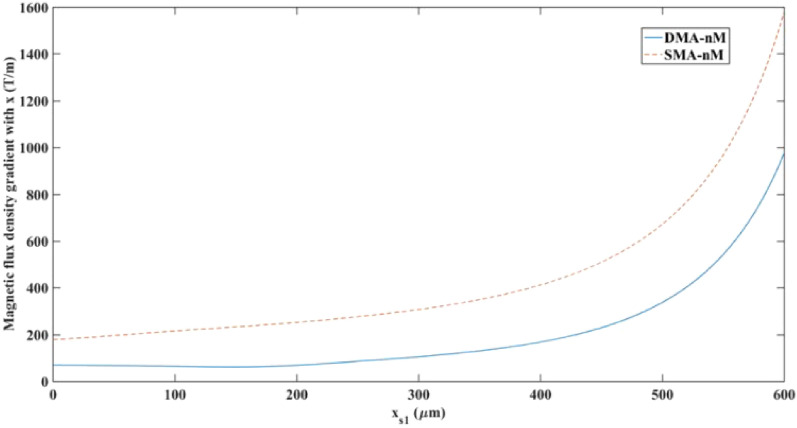
Magnetic flux density gradient in the x-direction along cutline xs1 and y = 0 [μm] in the separation region for the nM systems.

Figure 14 illustrates the created magnetic field in the x-direction, and it can be observed that the high local magnetic field gradient regions around the wire were created near the separation chamber with noticeably higher values in the SMA-nM. *B*_*y*_, in the nM scenario, was almost zero, while *B*_*x*_ distribution increased along the channel centerline due to the ferrofluid magnetization, as depicted in Fig. 15. The magnetic field was higher before the separation region in the DMA-nM, but the SMA-nM demonstrated higher positive gradients due to the local attractive regions. These gradients are better explored in Fig. 16.

Overall, the SMA-nM showed higher magnetic properties because the magnetization of the magnet is exerted along the x-axis, which directly magnetizes the ferromagnetic wire. Finally, it can be concluded from the study of the pM and nM modes that the magnetic gradient created by the wire was the primary cause of the interaction with the particles during the separation process.

### Particle trajectories

**Figure 17:**
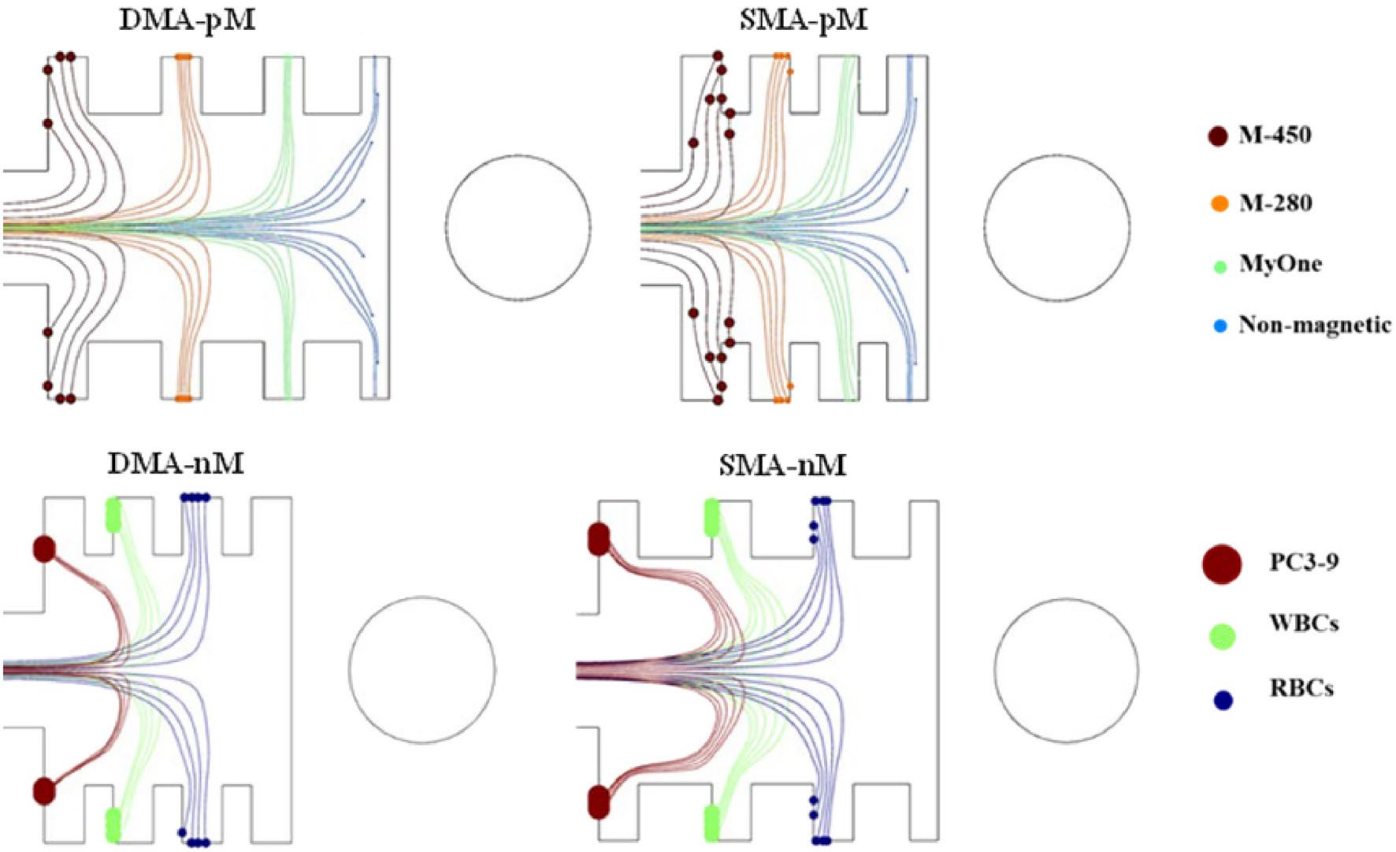
Particles' trajectories show the complete separation of the magnetic and biological targets in the pM and nM modes, respectively. The wire is kept at 100 [μm] from the separation chamber (edge-to-edge).

Figure 17 illustrates the simulated particles' trajectories for all the injected particles at the four configurations. A complete sorting of the large-sized M-450, the middle-sized M-280, and the smallest MyOne beads was achieved after tuning the inlet buffer velocities to 5 [mm/s] and 2 [mm/s] for DMA-pM and SMA-pM, respectively, while keeping the inlet sample velocity at 0.5 [mm/s] for both systems. Complete separation was also achieved for the RBCs, WBCs, and PC3-9 cells in the nM systems but with larger throughputs. Each particle exits directly to an outlet designed to correspond with its deflection trajectory induced by the repulsive forces according to its distinctive magnetic response. More importantly, these concept systems allow for increasing the throughput by increasing the cross-sectional flow area with the length of extended wire while keeping an efficient simultaneous separation of entities. Another great advantage is the possibility of tunable separation driving forces by moving the wire and manipulating the hydrodynamic focusing, which allows for handling different-sized particles in both modes.

### Effect of inlet velocities

**Figure 18:**
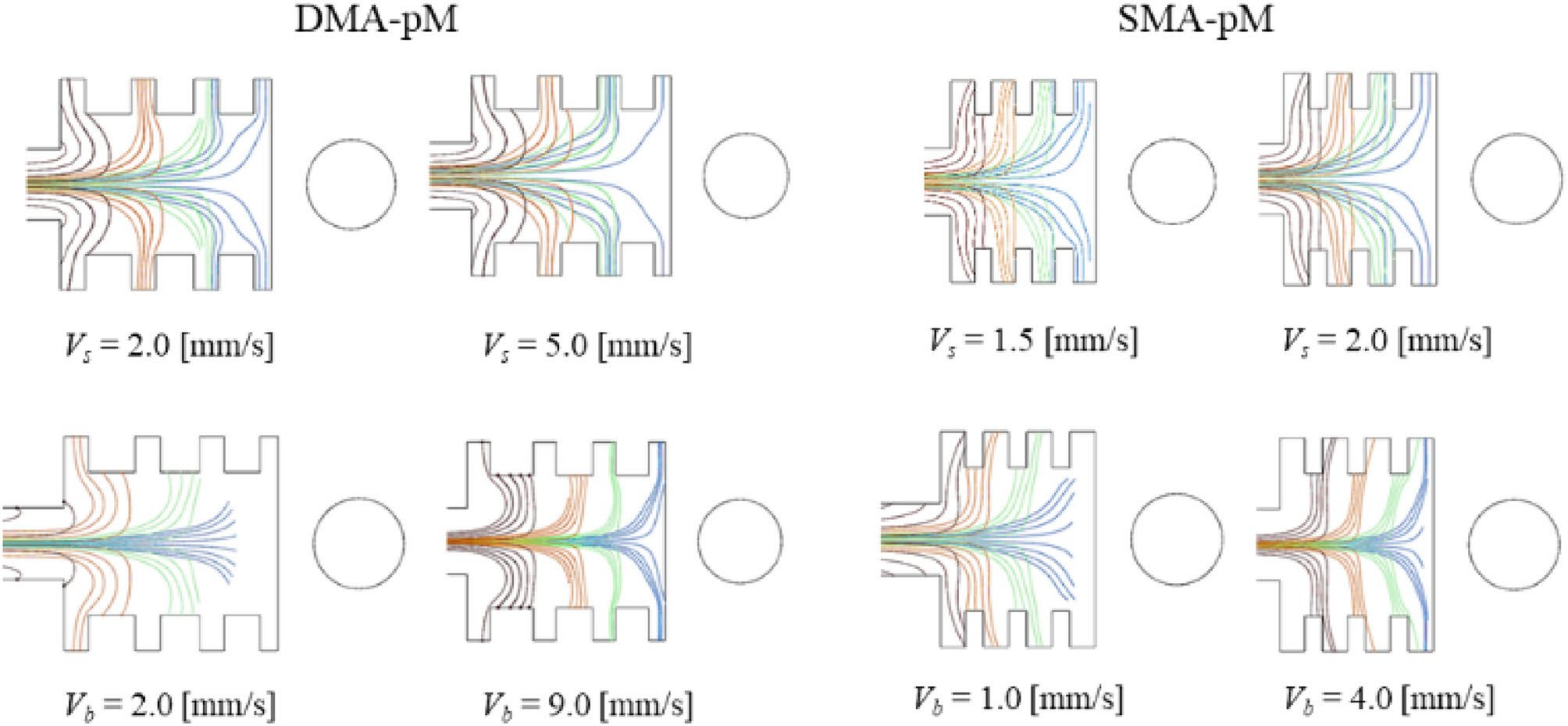
The impact of changing the inlet velocities for the sample (*V*_*s*_) and buffer (*V*_*b*_) on the particles' trajectories while keeping the wire at 100 [μm] from the separation chamber (edge-to-edge).

Although it is desirable to have high throughput from a sorting system, *V*_*s*_ and *V*_*b*_ are critical to achieving an efficient separation. Figure 18 shows that particles were being scattered as a result of injecting the sample with high velocities; *V*_*s*_: 2.0 [mm/s] and 5.0 [mm/s] in DMA-pM and 1.5 [mm/s] and 2.0 [mm/s] in SMA-pM, or by adopting low buffer flows, *V*_*b*_: 2.0 [mm/s] in DMA-pM and 1.0 [mm/s] in SMA-pM, which are not sufficient to focus the sample. On the other hand, too high buffer velocities, *V*_*b*_: 9.0 [mm/s] in DMA-pM and 4.0 [mm/s] in SMA-pM, pushed particles too much away from their distinctive outlets. In the previous studies, only one parameter was changed at a time, while all other parameters remained constant compared to the baseline cases.

### Effect of the wire distance from the microchannel

**Figure 19:**
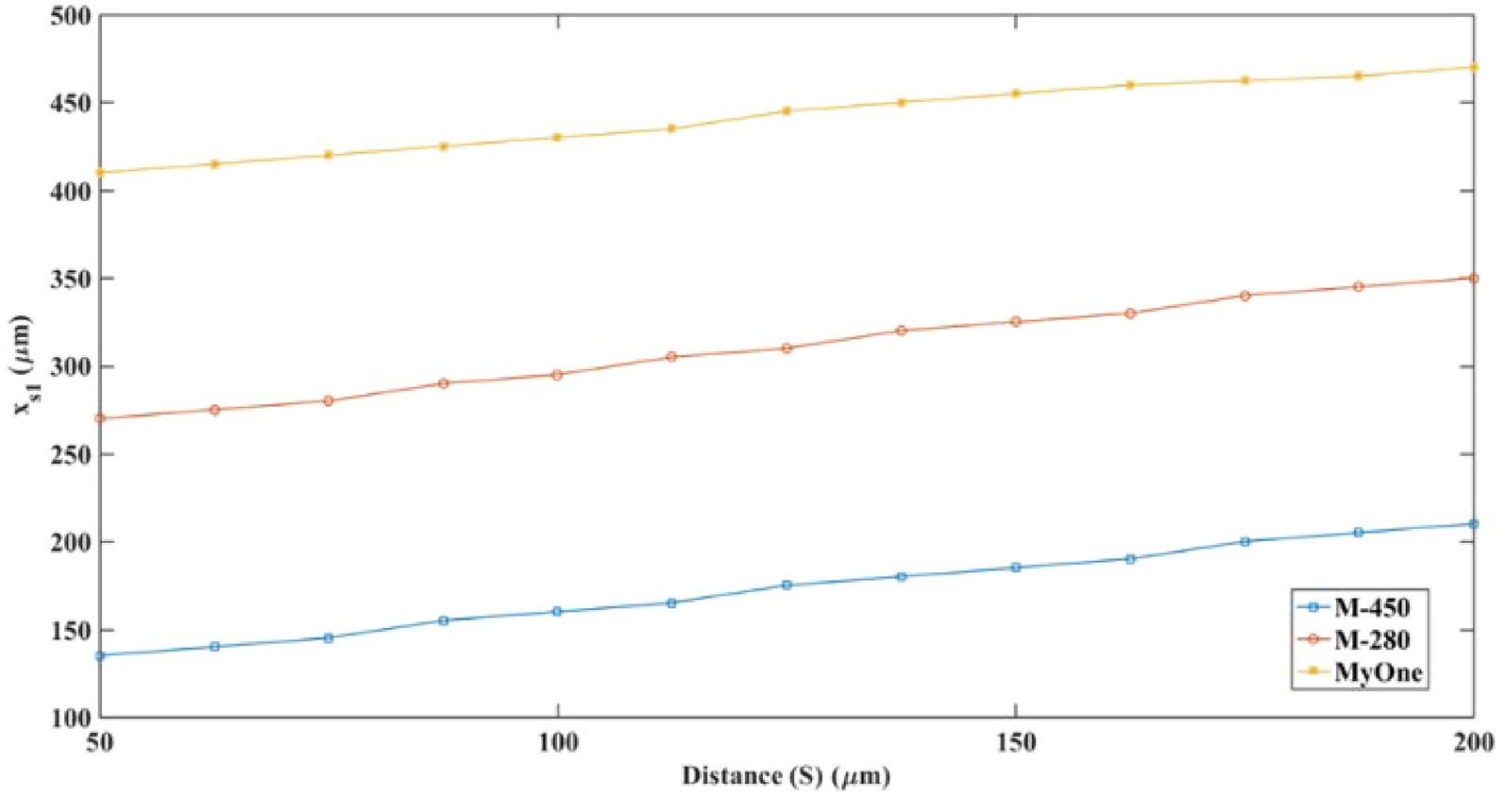
The impact of varying distance S on the separation point of three magnetic beads in the sorting region along cutline x_s1_ and y = 0 [μm].

A parametric study was done for the magnetic beads in the DMA-pM to determine how changing the distance S affected the separation point along the x_s1_. Figure 19 shows the estimated separation points along x_s1_ for the M-450, M-280, and MyOne particles at different S values ranging from 50 [μm] to 200 [μm]. The study was conducted using the same parameters as the DMA-pM. The figure shows the shift of the separation points of the M-450, M-280, and MyOne particles by 75 [μm], 80 [μm], and 60 [μm], respectively, as S increased from 50 [μm] to 200 [μm]. This shift can be interpreted by the decrease of the magnetophoretic forces affecting the bead's deflection as the repulsive field is formed over a limited angular vicinity of the wire. By adjusting the distance S, an automated device that can handle various types of beads based on their magnetic interaction can be created; the plots showed linear change. In a previous study by Khashan et al.^59^, the unfocused sample flow rate variation was investigated experimentally against the separation distance for a 4.3 [μm] particle, and the generated plot showed an exponential behavior of change. The study revealed the importance of hydrodynamic focusing on sorting efficiency and performance.

### Effect of expanding the separation chamber

The separation efficiency was studied for magnetic beads M-450 and M-280 at various values of the expansion ratio (ER), the ratio between the main channel and separation chamber widths. The results are shown in Table 3, while the MyOne bead was excluded as its separation efficiency was always 100%. In all the results presented in the table, the inlet sample velocity is maintained at 0.5 mm/s, and the inlet buffer velocity is at 5 mm/s. The repulsive force acting on the magnetic beads decreases as the wire moves away from the sorting chamber. However, the expansion of the separation region compensates for this decrease by reducing the drag resistance on the magnetic beads, resulting in improved separation efficiencies. At ER = 0.5, the value in the base case scenarios, the efficiency reached 100% for both magnetic beads when the spacing between the wire and the separation region (S) equaled 50 μm and 100 μm, while it was 75% for S = 150 μm. The efficiency could not reach 100% because the reduction in repulsive magnetic force caused by moving the wire away from the separation region cannot be fully compensated by the expansion effect.

**Table 3:** Summary separation efficiency values at different expansion ratio (ER) values.

S^a^	M-450				M-280			
	ER^b^ = 1	ER = 0.66	ER = 0.5	ER = 0.4	ER = 1	ER = 0.66	ER = 0.5	ER = 0.4
50	75	100	100	100	100	100	100	100
100	50	75	100	100	50	75	100	100
150	50	50	75	75	25	50	75	75

^a^$S$ is the spacing between the wire and the separation region [μm].

^b^$ER$ is the expansion ratio.

Efficiency was determined by tracking the specific exit of each bead type from its designated outlet. For instance, the efficiency for M-450 beads is deemed 100% if all M-450 beads are introduced at the inlet exit exclusively through their designated outlet. This method was similarly applied to calculate the efficiencies for MyOne beads and M-280 magnetic beads.

**Figure 20:**
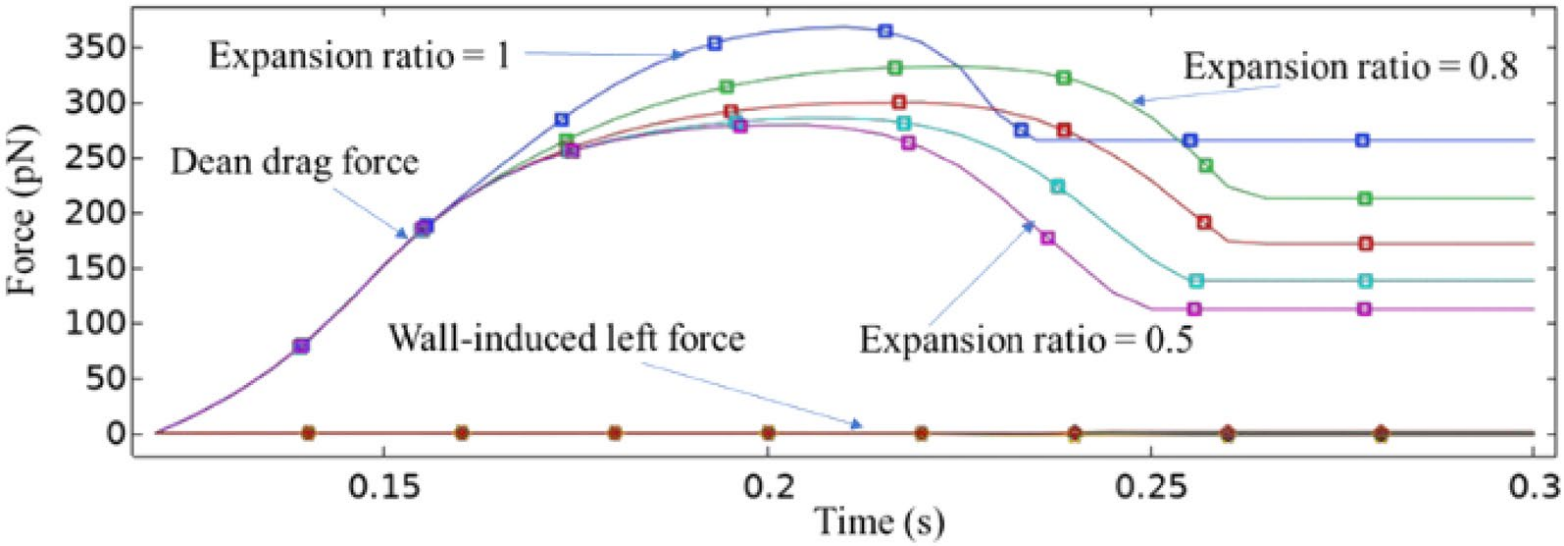
The impact of varying the expansion ratio of the separation chamber on the values of the Dean drag and wall-induced left forces.

Figure 20 illustrates how changing the ER affects the Dean drag and wall-induced left forces, which depend on fluid velocity and pressure. As the cross-sectional area of the separation chamber increases, the fluid velocity decreases, leading to a reduction in the Dean drag force. The wall-induced lift force is insignificant compared to the Dean drag force because of hydrodynamic focusing and multiple outlets in the separation chamber walls. When the ER is equal to 1, indicating no expansion or contraction, channel width remains uniform. This uniformity results in a more stable flow profile after the magnetophoretic effect occurs. Such stability leads to an earlier reduction in Dean drag forces, attributable to the absence of flow velocity and pressure variations that typically arise from changes in channel dimensions. Consistent channel geometry directly influences fluid dynamics, underscoring the importance of precise control over these factors in microfluidic systems, which are crucial for optimizing device performance.

**Figure 21:**
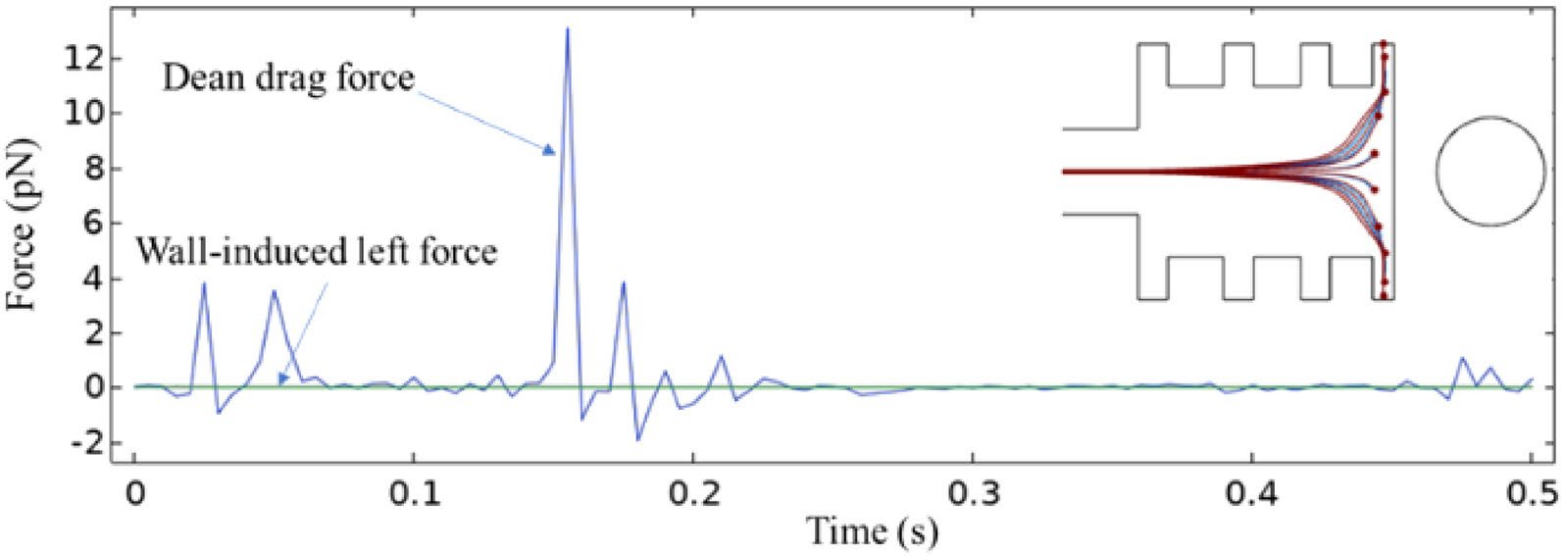
The Dean drag and wall-induced left forces plot with deactivated magnetophoretic force created by the ferromagnetic wire.

A simulation with deactivated magnetophoretic forces was performed to demonstrate the significance of the magnetic force generated by the magnetic arrangement, as shown in Fig. 21. The results indicated that the Dean drag and wall-induced left forces had a negligible effect on particle sorting, and it was evident that the driving force was the magnetic force created by the wire. In addition, it is noteworthy that the deactivation of the magnetic force led to a significant reduction in the Dean drag force compared with the case shown in Fig. 21.

**Figure 22:**
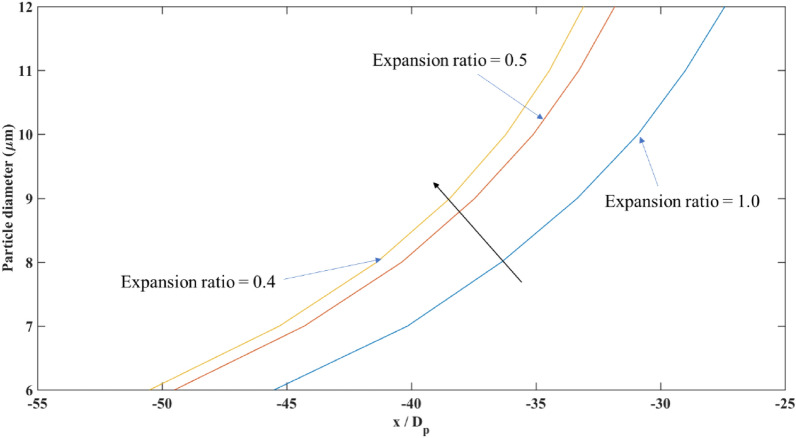
The particle’s size sensitivity against the longitudinal trajectories at different expansion ratios.

The effect of expanding the separation chamber on the size of injected particles was investigated through simulations. Figure 22 demonstrates the dimensionless longitudinal distance from the center of the wire at the separation chamber outlet versus the particle's diameter (Dp). As the expansion of the separation chamber increased, the distance between trajectories of different sizes increased. This is due to the decrease in magnetic field gradient, causing the trajectories of particles to spread out and follow wider paths. The sensitivity of the separation performance on the particle size was also investigated, with larger particles exhibiting a larger separation distance due to the interplay between the magnetic force, particle size, and magnetic field gradient. Understanding these factors is crucial for optimizing the design and efficiency of magnetophoretic separators for various applications.

## Conclusions

The magnetic characteristics and separation performance of two dual-mode transverse magnetic arrangements were investigated numerically. The two configurations may separate multi-magnetic and -biological targets using the pM and nM methods. The numerical analysis included studying the generated magnetic fields and hydrodynamics and examining the magnetophoretic and drag forces acting on microparticles of varied sizes and properties. The plots of the particles' trajectories showed a complete separation of the injected entity samples in all systems. In the pM modes, DMA-pM showed higher magnetic gradient generation and throughput for separating three magnetic and nonmagnetic particles than SMA-pM. In the nM modes, however, SMA-nM exhibited much higher magnetic properties and throughput than DMA-nM for sorting a sample containing RBCs, WBCs, and PC3-9 cells. The optimized hydrodynamic focusing of the sample flow affects the proper deflection of particles into their distinctive outlets. A parametric study revealed that the linear movement of the wire is a key parameter that can be utilized to have a tunable device. In addition, controlling the repulsive forces may lead to creating an automated separation device, which can handle a larger number of targets or produce customized separation microchannels based on the desired sample size by configuring the outlets' positions. On top of that, the device's throughput can be effectively increased compared with other devices, thanks to the transverse arrangement of the wire and the magnets, which allows for maximizing the pumped sample by increasing the microfluidic channel depth. The proof provided by the simulation is validated through experimental verification using positive magnetophoresis and DMA arrangement and employing polystyrene-based magnetic beads of varying sizes and magnetic content, along with nonmagnetic polystyrene beads. The results obtained from the fabricated microdevice demonstrated the capability to sort, switch, and separate microparticles continuously.

## Data Availability

The data that support the findings of this study are available on request from the corresponding authors.
